# Cluster K Mycobacteriophages: Insights into the Evolutionary Origins of Mycobacteriophage TM4

**DOI:** 10.1371/journal.pone.0026750

**Published:** 2011-10-28

**Authors:** Welkin H. Pope, Christina M. Ferreira, Deborah Jacobs-Sera, Robert C. Benjamin, Ariangela J. Davis, Randall J. DeJong, Sarah C. R. Elgin, Forrest R. Guilfoile, Mark H. Forsyth, Alexander D. Harris, Samuel E. Harvey, Lee E. Hughes, Peter M. Hynes, Arrykka S. Jackson, Marilyn D. Jalal, Elizabeth A. MacMurray, Coreen M. Manley, Molly J. McDonough, Jordan L. Mosier, Larissa J. Osterbann, Hannah S. Rabinowitz, Corwin N. Rhyan, Daniel A. Russell, Margaret S. Saha, Christopher D. Shaffer, Stephanie E. Simon, Erika F. Sims, Isabel G. Tovar, Emilie G. Weisser, John T. Wertz, Kathleen A. Weston-Hafer, Kurt E. Williamson, Bo Zhang, Steven G. Cresawn, Paras Jain, Mariana Piuri, William R. Jacobs, Roger W. Hendrix, Graham F. Hatfull

**Affiliations:** 1 Pittsburgh Bacteriophage Institute and Department of Biological Sciences, University of Pittsburgh, Pittsburgh, Pennsylvania, United States of America; 2 Department of Biological Sciences, University of North Texas, Denton, Texas, United States of America; 3 Department of Biology, Calvin College, Grand Rapids , Michigan, United States of America; 4 Department of Biology, Washington University, St. Louis, Missouri, United States of America; 5 Department of Biology, College of William and Mary, Williamsburg, Virginia, United States of America; 6 Department of Biology, James Madison University, Harrisonburg , Virginia, United States of America; 7 Department of Microbiology and Immunology, Albert Einstein College of Medicine, New York, New York, United States of America; Charité-University Medicine Berlin, Germany

## Abstract

Five newly isolated mycobacteriophages –Angelica, CrimD, Adephagia, Anaya, and Pixie – have similar genomic architectures to mycobacteriophage TM4, a previously characterized phage that is widely used in mycobacterial genetics. The nucleotide sequence similarities warrant grouping these into Cluster K, with subdivision into three subclusters: K1, K2, and K3. Although the overall genome architectures of these phages are similar, TM4 appears to have lost at least two segments of its genome, a central region containing the integration apparatus, and a segment at the right end. This suggests that TM4 is a recent derivative of a temperate parent, resolving a long-standing conundrum about its biology, in that it was reportedly recovered from a lysogenic strain of *Mycobacterium avium*, but it is not capable of forming lysogens in any mycobacterial host. Like TM4, all of the Cluster K phages infect both fast- and slow-growing mycobacteria, and all of them – with the exception of TM4 – form stable lysogens in both *Mycobacterium smegmatis* and *Mycobacterium tuberculosis*; immunity assays show that all five of these phages share the same immune specificity. TM4 infects these lysogens suggesting that it was either derived from a heteroimmune temperate parent or that it has acquired a virulent phenotype. We have also characterized a widely-used conditionally replicating derivative of TM4 and identified mutations conferring the temperature-sensitive phenotype. All of the Cluster K phages contain a series of well conserved 13 bp repeats associated with the translation initiation sites of a subset of the genes; approximately one half of these contain an additional sequence feature composed of imperfectly conserved 17 bp inverted repeats separated by a variable spacer. The K1 phages integrate into the host tmRNA and the Cluster K phages represent potential new tools for the genetics of *M. tuberculosis* and related species.

## Introduction

Bacteriophages represent a numerical majority of biological entities in the biosphere although their full genetic diversity remains ill-defined [1]. Many different virion morphologies have been described, with the largest group being the *Caudovirales*, double-stranded DNA (dsDNA) tailed phages [2]. The complete sequences of approximately 750 phage genomes have been determined, although over 70% of the sequenced dsDNA genomes correspond to just twelve bacterial hosts [1], [3]. As many or more prophages also have been identified in bacterial genome sequencing projects [4], [5]. Although phages of different bacterial hosts typically share little nucleotide sequence similarity, phages of common hosts can also represent substantial diversity at the nucleotide level [6], [7].

Comparative analysis of 80 mycobacteriophage genomes reveals substantial but not homogenous diversity. Although many phages have little or no sequence similarity to each other, examples of genomes with substantial nucleotide sequence similarities have also been documented [7], [8]. To facilitate analysis, the phages have been sorted into clusters on the basis of gross DNA relationships using the cluster metrics described previously [6], [9], and a total of eleven Clusters (A–K) have been described [8]. The diversity among phages within clusters varies greatly. At one extreme, the four genomes of Cluster G – Angel, BPs, Halo and Hope – differ in only few nucleotide positions and at the variable position of the Mycobacteriophage Mobile Element (MPME) [8], [10]. At the other extreme, the current members of both Clusters A and B can be subdivided into four subclusters (A1–A4, B1–B4), with subclustered genomes having common genomic architectures but relatively low levels of nucleotide sequence similarity [8]. Five of the 80 completely sequenced mycobacteriophage genomes – Corndog, Giles, LeBron, Omega, and Wildcat – are ‘singletons’, having no close relatives [8]. There are a total of 26 different subcluster and singleton genomes, a remarkably large number for a collection of phages that infect a common bacterial host strain, *M. smegmatis* mc^2^155. Like most phage genomes, mycobacteriophages have mosaic genomic architectures [9], [11] with illegitimate recombination predicted to play a key role in the exchange of modules amongst phage types [12].

Mycobacteriophages provide extremely useful tools for the study and manipulation of their hosts. Many mycobacteriophages were isolated originally for uses in phage typing of clinical bacterial isolates [13], [14] but have since proven to be workhorses for developing mycobacterial genetics. A landmark achievement was the construction of shuttle phasmids – chimeric vectors that replicate in *Escherichia coli* as cosmids and upon transfection of *M. smegmatis* yield mycobacteriophage particles that can deliver foreign DNA to Bacillus Calmette Guérin (BCG) or *M. tuberculosis* [15]. Incorporation of a drug resistance marker into a temperate shuttle phasmid led to the development of the first transformation systems [16], and addition of reporter genes such as firefly luciferase or green fluorescent protein enabled construction of tools for rapid diagnosis and drug susceptibility testing of *M. tuberculosis* [17], [18], [19]. Other applications include the efficient delivery of transposons to generate transposon libraries [20], [21], [22], and for targeted gene replacement [23] or transfer of point mutations [24] by specialized transduction. Mycobacteriophages have also been adapted for diagnostic applications in amplification assays [25], [26], [27] and exploited for the development of integration-proficient vectors [28], [29], non-antibiotic selectable markers [30], and recombineering systems [31], [32], [33], [34].

Mycobacteriophage TM4 plays a central role in mycobacterial genetics, being the first phage to be used for shuttle phasmid construction [15] and still widely employed for efficient gene delivery to *M. tuberculosis*. It has also been useful for understanding the role of phage-encoded WhiB proteins [35], lysis systems [36], and the role of conserved peptidoglycan hydrolyzing motifs in tapemeasure proteins [37]. The phage was initially recovered from a putative lysogenic strain of *M. avium* [38], although following purification the original host strain was not immune to TM4 superinfection [38]. It has a broad host range infecting both fast- and slow-growing mycobacteria [39] but does not appear to form stable lysogens in any strain [15], [17]. Timme *et al.*, (1984) suggest that either the original strain became cured of its prophage, or the prophage is present in a pseudolysogenic state, such that a majority of cells remain susceptible to infection. The complete sequence of the TM4 genome [40] shows that it is 52,797 bp in length and contains 92 predicted protein-coding genes and no tRNA genes [40]. There is no evidence for a phage repressor, but because repressors encompass considerable sequence diversity [30] they cannot always be readily identified bioinformatically. It is clear that TM4 does not encode a serine- or tyrosine-integrase, partitioning functions, or recognizable transposases that might indicate a temperate life-style. Thus the relationship of TM4 to its parent strain *M. avium* 8/9 serovar 4 remains unresolved.

A potential disadvantage of the use of shuttle phasmids for gene delivery purposes is that infection typically results in phage replication and lysis of the infected host. To circumvent this, conditionally replicating derivatives of TM4 have been isolated that grow at 30°C but fail to replicate at 37°C [20], [41]. To ensure that reversion to temperature resistance does not interfere with recovery of derivatives at 37°C that require low frequency events (such as transposon or recombination), conditionally replicating derivatives such as TM4 derivative ph101 were generated using multiple rounds of mutagenesis; these presumably contain two more mutations that contribute to the phenotype [20]. Similar TM4 shuttle phasmids have also proven useful for gene delivery in *Mycobacterium marinum* [42], [43] and *Mycobacterium avium subsp. Paratuberculosis* [44], [45].

We recently described the isolation of two phages – Angelica and CrimD – with discernible nucleotide sequence similarities to TM4. The three phages formed a new cluster, Cluster K, and are divided into two subclusters, K1 (Angelica and CrimD) and K2 (TM4) according to their nucleotide relationships [8]. Here we report the discovery of three additional Cluster K phages, Anaya, Adephagia, and Pixie, and provide a detailed comparative analysis of the Cluster K genomes. Interestingly, all of these phages – with the notable exception of TM4 – are temperate and form stable lysogens in both fast- and slow-growing mycobacteria. The integration functions are identified, but appear to be deleted from the center of the TM4 genome. These observations suggest that TM4 has undergone relatively recent deletion events that explains its biological oddities. We also map the mutations that give rise to the conditionally replicating phenotype in the TM4 derivative ph101.

## Results and Discussion

### Phage isolation and genome sequencing

The isolation of TM4, Angelica, and CrimD has been described previously [8], [38], as well as their genomic sequences [8], [40]. Phages Anaya and Adephagia were isolated at Calvin College and the University of North Texas respectively as part of a freshman research-based course supported by the Howard Hughes Medical Institute (HHMI) Science Education Alliance (SEA). Pixie was isolated at the University of Pittsburgh as part of its Phage Hunters Integrating Research and Education (PHIRE) program [46]. All were isolated by the plating of environmental samples on lawns of *Mycobacterium smegmatis* mc^2^155; Pixie, Adephagia, and Anaya were recovered after enrichment by growth in the presence of *M. smegmatis*.

Following plaque purification, DNA was isolated, and each genome was shotgun sequenced using 454 technology to at least 25-fold redundancy (∼4000 to ∼8000 reads per genome). In the cases of Adephagia and Anaya, shotgun Illumina reads (100-fold redundancy) were also generated to strengthen any weak points in the 454 data. Remaining ambiguities and the nature of the genome termini were resolved by targeted Sanger sequencing with oligonucleotide primers using phage genomic DNA as a template. The general genomic features of these phages are shown in Table 1.

**Table 1:** Genometrics of Cluster K mycobacteriophages.

Phage	Length (bp)	Overhang	# ORFs	# tRNAs	School	Location	Subcluster	Accession #
Adephagia	59,646	11 bases	94	1	UNT	Denton, TX	K1	JF704105
Anaya	60,835	11 bases	98	1	Calvin	Grand Rapids, MI	K1	JF704106
Angelica	59,598	11 bases	94	1	WUSTL	University City, MO	K1	HM152764.1
CrimD	59,798	11 bases	95	1	Wm. & Mary	Williamsburg, VA	K1	HM152767.1
TM4	52,797	10 bases	91	0	–	Denver, CO	K2	AF068845.1
Pixie	61,147	11 bases	100	0	Pitt	Houston, TX	K3	JF937104

### Cluster and subcluster assignments

**Figure 1:**
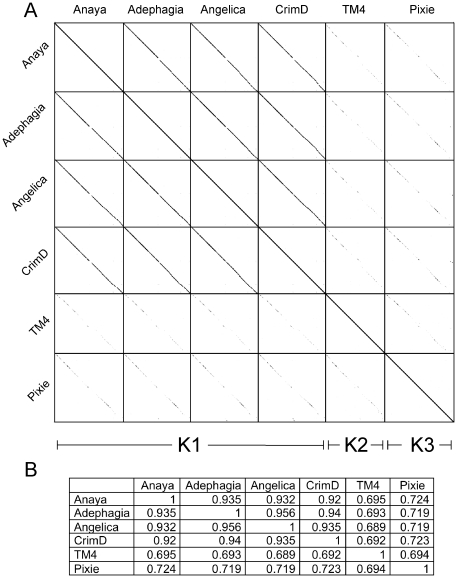
Dotplot comparison of Cluster K genomes. **A**. Nucleotide sequences of Cluster K genomes were concatenated and compared to themselves and each other using the dotplot generator Gepard [75]. Phages Adephagia, Anaya, Angelica, and CrimD show extensive nucleotide identity to each other while TM4 and Pixie are less similar, supporting division into Subclusters K1, K2 and K3 as shown. **B**. Average nucleotide identities of Cluster K mycobacteriophages.

Comparison of the genome sequences of phages Anaya, Adephagia, Angelica, CrimD, and Pixie by dotplot analysis shows that they share extensive nucleotide sequence similarity (Fig. 1A). This similarity is clearly to different degrees, but none show substantial DNA similarity to any other sequenced mycobacteriophages (data not shown). Anaya, Adephagia, Angelica and CrimD show strong similarity (>92% pair wise average nucleotide similarity; ANI, Fig. 1B) and constitute Subcluster K1. Both TM4 and Pixie share less than 73% ANI to the other phages in that TM4 constitutes Subcluster K2 [8], and Pixie is the sole member of the new Subcluster, K3 (Fig. 1).

### Virion morphologies

**Figure 2:**
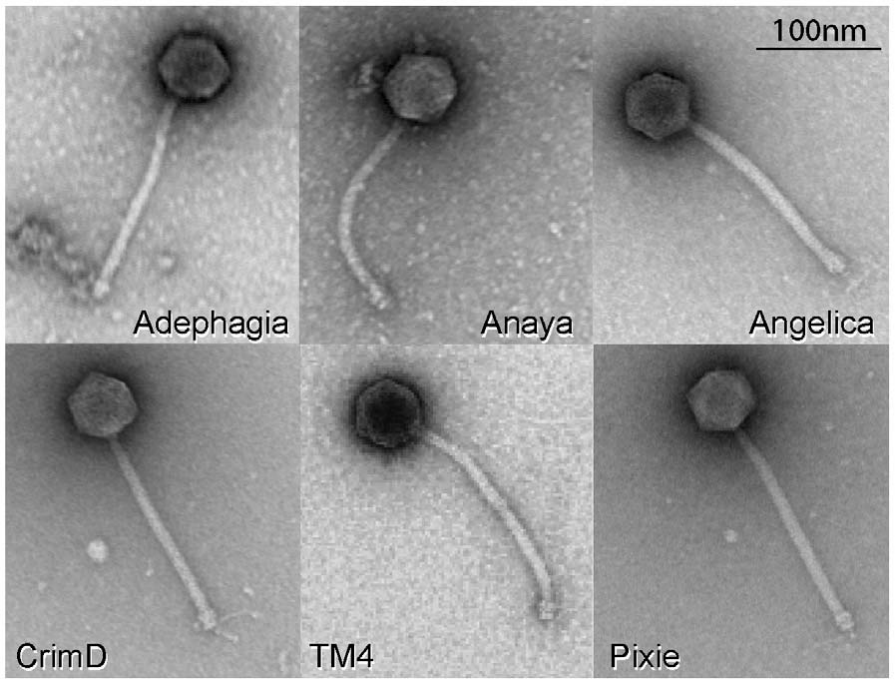
Virion morphologies of Cluster K phages. Particles of Cluster K phages were put on 400 mesh coated copper grids and stained with 1% uranyl acetate. Virions were imaged using a Morgagni transmission electron microscope. All the cluster K phages exhibit a flexible non-contractile tailed morphology with short side tail fibers. Virion capsids are ∼55 nm in diameter and tails average ∼190 nm in length.

Electron microscopy of the Cluster K phages shows that they have similar particle morphologies with long flexible non-contractile tails and isometric heads (Fig. 2). The heads of all six phages are approximately 55 nm in diameter and the tails are 185–200 nm long. The Cluster K phages are thus classified morphologically as members of the *Siphoviridae*. Short side tail fibers at the tip of the tail can be seen on many of the particles.

### Host-range of Cluster K phages

The host-range of TM4 has been described previously [38], [39], [45]; it is reported to infect fast-growing mycobacteria such as *M. smegmatis* as well as the slow-growing *M. tuberculosis H37Rv* and *M. ulcerans*. However, these reports differ in regards to the infection of *M. avium* by TM4, with substrains *M. avium* 701; 6, *M. avium* 702; 7, *M. avium* 3746/02 being resistant to infection [39], whereas substrains *M. avium* Bridge, serovar 2, *M. avium* 158, serovar 2, *M. avium* TMC 1419, serovar 2, and *M. avium* TMC 1461, serovar 2 are sensitive [45]. Timme et al (1984) report that TM4 infects nine *M. avium* strains, all of different serovars. Rybniker et al (2006) postulate that because TM4 was derived from a putative lysogenic strain of *M. avium* 6/8 serovar 4, the failure to infect some substrains of *M. avium* may be due to superinfection immunity conferred by resident prophages.

**Figure 3:**
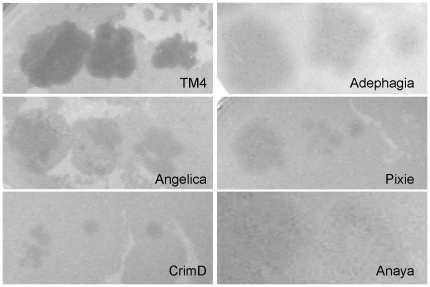
All Cluster K phages infect *M. tuberculosis*. Lysates of Cluster K phages were serially diluted with phage buffer and dilutions were spotted onto lawns of *M. tuberculosis* mc^2^7000. All cluster K phages infect *M. smegmatis* mc^2^155 (data not shown) and *M. tuberculosis* mc^2^7000 with equal efficiency. TM4 forms clear plaques whereas all other Cluster K phages form turbid plaques.

We tested phages Adephagia, Anaya, Angelica and CrimD as examples of Subcluster K1 as well as TM4 and Pixie for plaque formation on *M. tuberculosis* mc^2^7000, *M. bovis* BCG strain Pasteur, *M. avium* 104, and *M. marinum* strains M and 927. All six phages infected *M. tuberculosis* mc^2^7000 efficiently, albeit with different plaque morphologies (Fig. 3); TM4 yields larger clear plaques while Angelica, CrimD, and Pixie produce smaller, turbid plaques. Adephagia and Anaya produce large turbid plaques, although Anaya only produces plaques when incubated at or below 33°C. Only TM4 showed infectivity on *M. bovis BCG*, although we observed a reduction of efficiency of plating relative to *M. smegmatis* by between five and six orders of magnitude. No infectivity of *M. avium* 104 was observed with any of the Cluster K phages tested here. Adephagia, Anaya, Angelica and CrimD showed no infection of either *M. marinum* strain, although both TM4 and Pixie did, albeit at a greatly reduced efficiency of plating (data not shown). Plaques picked from these plates and re-spotted on lawns of *M. marinum* did not show an increased ability to infect either *M. marinum* strain over the parent phages. These plaques were only observed using 0.35% top agar and incubating at room temperature. We do not yet know the basis for these observed reductions in plating efficiencies, although it could be the result of restriction, CRISPR's, abortive infection, or the need for mutations that would expand the host range.

### Temperature-dependence of Anaya growth

Despite its genome sequence similarity to the other members of Cluster K1, Anaya does not share the same temperature growth range. No wild-type Anaya plaques were observed on *M. smegmatis* or *M. tuberculosis* lawns when plates were incubated at temperatures higher than 33°C; however, it was possible to isolate stable high-temperature resistant Anaya mutants from high titer *M. smegmatis* infections at 37°C. A wild-type Anaya lysate incubated at 37°C for one hour retained infectivity, indicating that the particles themselves do not dissociate at elevated temperatures. The nature of this temperature sensitivity during infection remains unclear.

### Anaya, Adephagia, Angelica, CrimD, and Pixie are temperate phages

It has previously been reported that TM4 behaves as a lytic phage in infection of *M. smegmatis* or *M. tuberculosis*, and lysogens have not been reported [17]. Furthermore, the genome of TM4 contains no readily identifiable features to suggest that it is competent to form lysogens [40]. However, during the host range analyses described above, it was evident that all of the other Cluster K phages form turbid plaques on all the susceptible strains tested. The Cluster K1 phages consistently show uniform, medium-sized plaques (∼2 mm dia.), although Pixie plaques are smaller, with more variation in size and less turbidity. Using these phages, we successfully recovered lysogenic derivatives of *M. smegmatis* that both confer immunity to self-superinfection, and release phage spontaneously into culture supernatants. Integration of the genomes was confirmed by PCR across one of the putative attachment junctions (see below). In spite of further attempts, we were unsuccessful in recovering any TM4 lysogens.

**Figure 4:**
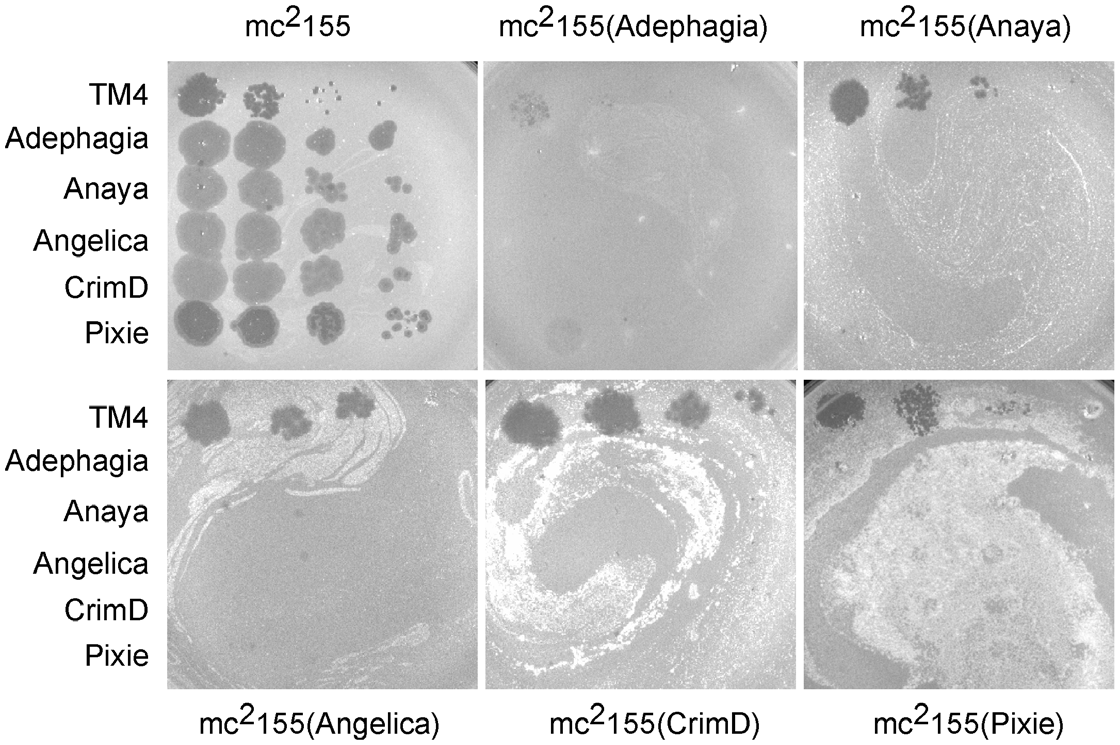
Immune specificities of Cluster K phages. Cluster K phages were serially diluted 100-fold and spotted onto lysogens of Subcluster K1 and K3 phages, as shown.

We have determined the immune specificities of each of the Cluster K phages (Fig. 4). Interestingly, we observed patterns of reciprocal immunity of the K1 and K3 phages, and presumably this homoimmune group of phages has related repressor-operator systems. In contrast, TM4 efficiently infects all of the lysogenic strains tested (Fig. 4). We note that the Adephagia lysogen behaves somewhat differently to the other K1 phages and appears to confer at least partial immunity to all of the phages tested, including TM4 and Pixie (Fig. 4). These observations are especially revealing about TM4 and its previously characterized properties. A simple explanation is that TM4 is a relatively recent derivative of a temperate phage that was heteroimmune with other Cluster K phages, but which has lost its immunity functions. When this event may have happened is unclear, it could have occurred during passage of the phage between its isolation in 1984 and genome sequencing in 1998, during the process of isolation, or at some prior time as a naturally occurring event. This is discussed in greater detail below. We note the obvious parallels to the relationship between D29 and Cluster A phages such as L5 [47], [48]. In D29, a 3.6 kbp deletion removes a segment that in L5 contains the repressor, and although D29 is lytic in nature, it is homoimmune with L5 immunity [30].

### Revisions to the TM4 genome annotation

**Figure 5:**
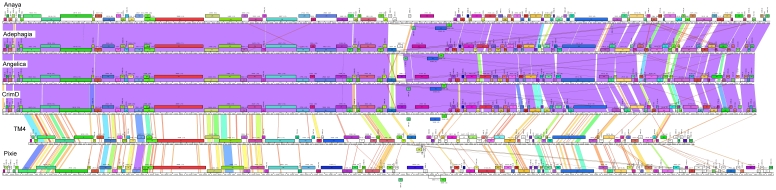
Global comparison of Cluster K genomes. A database of 83 sequenced mycobacteriophages (Mycobacteriophage_83) was analyzed using the program Phamerator (S. Cresawn, RHW & GFH, manuscript submitted) and used to compare the genome organizations of the six Cluster K phages. The top four genomes (Anaya, Adephagia, Angelica and CrimD) constitute Subcluster K1 and their overall nucleotide similarities are reflected by the violet shading between the genomes (nucleotide similarities between adjacent genomes are spectrum color-coded with violet being the most similar, and red the least similar). TM4 and Pixie belong to Subcluster K2 and K3 and their more distant relationships are evident. Each of the genes (boxes above or below each genome) are colored according to their phamily designation and the shared genome organizations of all six phages is consistent with their grouping into Cluster K.

**Figure 6:**
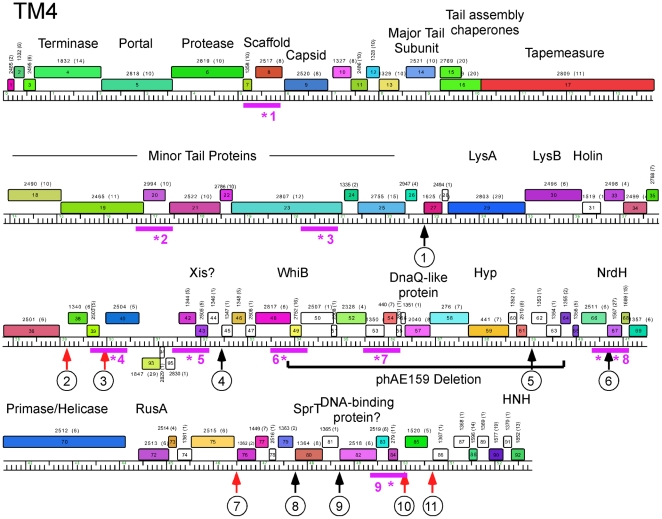
Genome map of Mycobacteriophage TM4. A map of the TM4 genome was revised from that reported previously [40]. The genome is shown with markers spaced at 100 bp intervals, with genes shown as colored boxes, either above (rightwards transcribed) or below (leftwards transcribed) the genome. Gene names are shown within the boxes, and the phamily number of that gene shown above with the number of phamily members in parentheses. Genes are colored according to their phamily, and white genes represent orphams (phams with only a single member). Genes *92*, *93*, and *94* are newly assigned, transcribed in the reverse direction from the rest of the genes of the genome, and replace gene *41* in the original TM4 annotation (see figure 11A). Putative gene functions are indicated. Also shown is a segment that is deleted in construction of shuttle phasmid phAE159 [23], and the locations of mutations (purple asterisks) and PCR amplicons (purple bars) used in their analysis. Vertical arrows with numbers show the positions of Start-Associated Sequences (SAS), either with (ESAS; red arrows) or without (black arrows) extended SAS sequences (see Figs. 14 and 15). SAS and ESAS sites are numbered as in Fig. 14 and are all in one orientation unless indicated otherwise with a minus sign.

**Figure 7:**
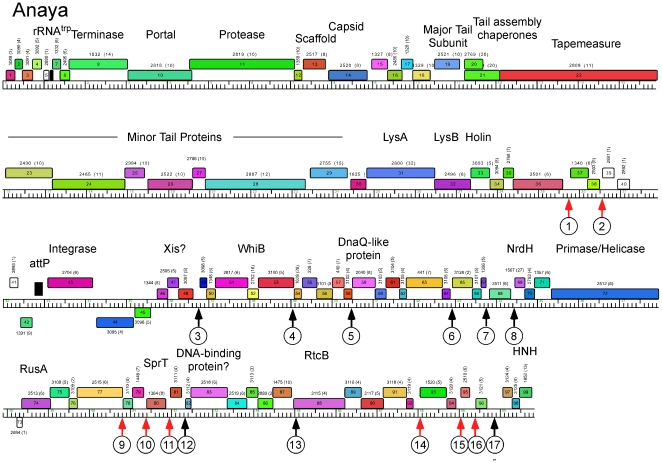
Genome map of Mycobacteriophage Anaya. The genome map of Anaya is shown with annotations as described for Figure 6.

**Figure 8:**
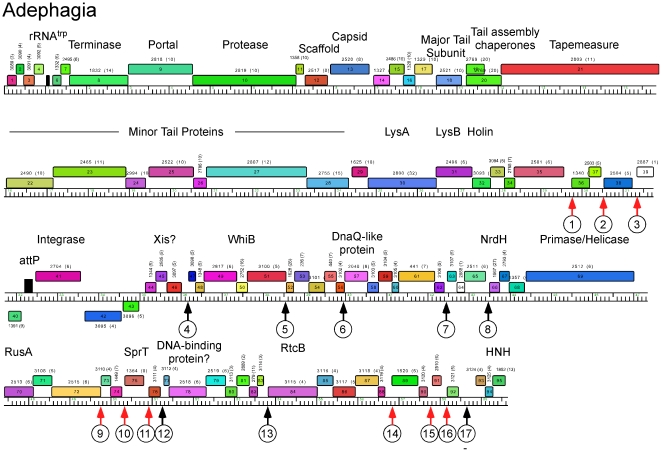
Genome map of Mycobacteriophage Adephagia. The genome map of Adephagia is shown with annotations as described for Figure 6.

**Figure 9:**
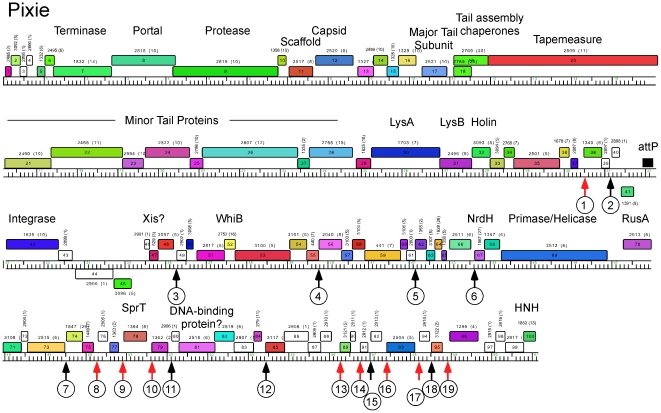
Genome map of Mycobacteriophage Pixie. The genome map of Pixie is shown with annotations as described for Figure 6.

**Figure 10:**
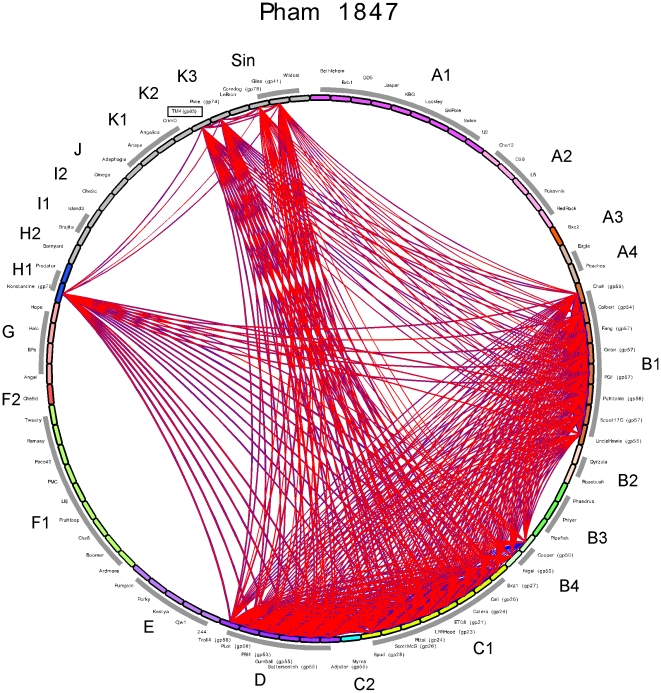
Phamily circle of Pham1847. A phamily circle of Pham1847 is shown with each of the 83 phages around the circumference of the circle and arcs drawn between phages that contain a member of Pham1847; BLASTP values shown as blue lines and ClustalW similarities as red lines.

The development of improved bioinformatic tools and the advantages of comparative genome analysis facilitate a revision of the TM4 genome annotation, an important consideration given its widespread use in mycobacterial genetics (Table S1; Figs. 5, 6, 7, 8, 9). We propose that three formerly identified orfs – designated as genes *32*, *37*, and *71*, are removed. The first two are very small (90 bp and 150 bp respectively) and show no compelling evidence of coding potential. The third (*71*) is somewhat larger (294 bp) but also shows little evidence of coding potential. In the central part of the genome, there was formerly a single small rightwards-transcribed orf (336 bp), designated gene *41*. We propose that this is replaced by three small orfs on the opposite strand, designated as genes *93*, *94* and *95* (to maintain the prior gene naming scheme). Although all three are small, they all show good coding potential as predicted by GeneMark [49]. In addition, relatives of *93* (Pham 1847) are present in Pixie (as gene *74*, Fig. 9) – although not in the Subcluster K1 phages – and distributed broadly among a diverse collection of different mycobacteriophages (Fig. 10). The closest relative of TM4 gp93 is Pixie gp74 although the proteins share only 53.7% identity, and the route by which TM4 gene *93* arrived at its current genomic location in TM4 is unclear.

In addition, there are five TM4 genes for which an alternative start codon is predicted, genes *12*, *26*, *51*, *66*, and *76*. Two of these were previously annotated to use either AUG (gene *76*) or GUG (gene *26*), and all have been re-annotated to use a UUG start codon, with better predictions for ribosome binding sites and better alignment with the predicted coding potential. Translation start sites for genes *12*, *51*, and *66* were changed to more closely reflect the coding potential (Table S1).

### Cluster K genome organizations

Five of the six Cluster K genomes (Anaya, Adephagia, Angelica, CrimD and Pixie) are of similar lengths (59.1–61.1 kbp) with TM4 (52.7 kbp) being approximately 7 kbp shorter (Table 1). All of the viral genomes are linear with defined ends having 3′ single-stranded DNA complementary extensions; all have 11-base extensions with the exception of TM4, which is reported to have a 10-base extension [40]. The genomes contain between 90 and 100 predicted protein-coding genes and the four Cluster K1 genomes – Anaya, Adephagia, Angelica and CrimD – all encode a single tRNA^trp^ near their left end (see Tables S1, S2, S3, S4, S5, S6).

To facilitate comparative genomic analysis, the three newly sequenced genomes were added to the 80 previously described mycobacteriophage genomes to create a database (Mycobacteriophage_83) for the genome comparison program, Phamerator [50]. The total of 9,308 predicted protein-coding genes were compared with each other using ClustalW and BlastP, and assembled into 2,667 phamilies using previously published parameters (manuscript submitted). Of these, 1,120 (47.3%) are orphams (phams containing only a single gene member). The mean pham size is 3.932.

An overview of the relationships between the six cluster K phages is shown in Fig. 5, and several patterns emerge. First, the extent of nucleotide sequence similarities between the genomes are clearly illustrated, and emphasizes the close similarity among the Cluster K1 phages, and the more distant relationships between these and the subcluster K2 and K3 phages. The left parts of the Cluster K1 genomes are especially closely related, with greater deviations in the right parts (Fig. 5). Secondly, the overall genome architecture is shared by all six phages with a substantial number of shared genes, as seen from the commonality of the color-coded pham assignments (Fig. 5). Thirdly, the basis for the smaller size of the TM4 genome compared to both its subcluster K1 and K3 relatives is apparent, with reductions in size near the left end, in the middle, and at the extreme right end (Fig. 5; see below).

Genome maps of Anaya, Adephagia, TM4 and Pixie are shown in Figures 6, 7, 8, 9 [Angelica and CrimD were reported recently [8] and maps are provided as Figs. S1 and S2; the TM4 map (Fig. 6) is a revision of that reported previously [40]. In all of the Cluster K phages the virion structure and assembly genes occupy the leftmost 22–24 kbp and are transcribed rightwards. There is considerable departure among the genomes at their extreme left ends, with a variable number of small genes of no known function between the terminase large subunit gene and the left physical end. All of the K1 phages, but neither TM4 nor Pixie, contain a tRNA^trp^ gene in this region. Within the virion structure and assembly genes there are a few notable differences between the genomes. First, the putative capsid assembly proteases of the K1 and K3 phages are larger than that of TM4 (Figs. 6, 7, 8, 9) due to a central insertion of about 1.1 kbp. This central portion does not appear to be related to inteins, homing endonucleases, or other mobile elements, but does have weak sequence similarity to parts of methyl-accepting chemotaxis proteins of several bacteria including *Planctomyces limnophilus* and *Chromobacterium violaceum*; however, it is compositionally biased (rich in alanine) which could account for the weak sequence similarity. The tapemeasure proteins are similar in length with the exception of Pixie gp20, which is 114 amino acid residues longer than the others; Pixie has a correspondingly longer tail than the other Cluster K phages (Fig. 2). To the right of the tail genes are the lysis cassettes, each of which contains a Lysin A gene, a Lysin B gene, and a putative holin gene. However, there is substantial diversity among the Cluster K phages in these genes. For example, the Lysin A of Pixie (gp31) is unrelated to the other Cluster K Lysin A proteins, and is more closely related to the Lysin A proteins of Cluster E phages (sharing, for example, 65% amino acid identity with Cjw1 gp32). The putative holin genes are downstream of the Lysin B genes, each containing 4–5 putative membrane-spanning domains and are only weakly related to each other and not across their entire spans. The 7–8 rightwards transcribed genes to the right of the lysis cassettes (e.g. Anaya genes *34*–*40*, Fig. 7) are of unknown function, although we note that Anaya gene *36* and its relatives in the other five Cluster K phages have relatives in distantly related phages including *Propionibacterium acnes* phage PA6. This region is one of the most diverse among the Subcluster K1 phages (Fig. 5).

With the exception of TM4 (see below), integration cassettes containing putative integrase genes and *attP* sites are located close to the center of the genomes; the integrases are of the tyrosine recombinase family and the *attP* sites are located to the 5′ side of the integrase genes (Figs. 7, 8, 9). The integration cassettes are flanked by a small number of genes transcribed in the leftwards direction, whose function is unknown. Putative Xis genes encoding proteins with MerR-like DNA binding domains are located to the right within an apparently long rightwards-transcribed operon that extends to the right end of the genomes. This region contains WhiB-related proteins, e.g. TM4 gp49, a protein that has been shown to be non-essential for TM4 growth [35] although it is well-conserved among the Cluster K phages. Other genes whose functions can be predicted from database similarity searches are those related to SprT (e.g. Pixie gp78), RusA (e.g. Adephagia gp75), HNH homing proteins (e.g. TM4 gp92), glutaredoxin-like NrdH proteins (e.g. TM4 gp67) and a large Primase/Helicase protein (e.g. TM4 gp70). The Subcluster K1 genomes also encode relatives of RtcB (e.g. Anaya gp88), a putative RNA ligase component [51]. Because only the Subcluster K1 genomes encode both tRNA and the RtcB proteins, we speculate that these phage-encoded RtcB proteins play a role in protection against a host-mediated tRNA cleavage defense against viral infection [52]. The remainder of the proteins encoded in these regions are of unknown function, and we note that about 30% of the Pixie genes in this region are orphams, reflecting its high genomic diversity from all other mycobacteriophages.

### TM4 is a derivative of a temperate parent

TM4 was originally isolated by recovery from a strain of *M. avium*, although understanding its origin is complicated by the observations that it is able to infect the original *M. avium* strain and does not appear to be temperate in any mycobacterial host [39] (Fig. 3). Because the related Cluster K phages are all temperate, we have investigated potential genes that are deleted in TM4 and that could contribute to a temperate lifestyle.

**Figure 11:**
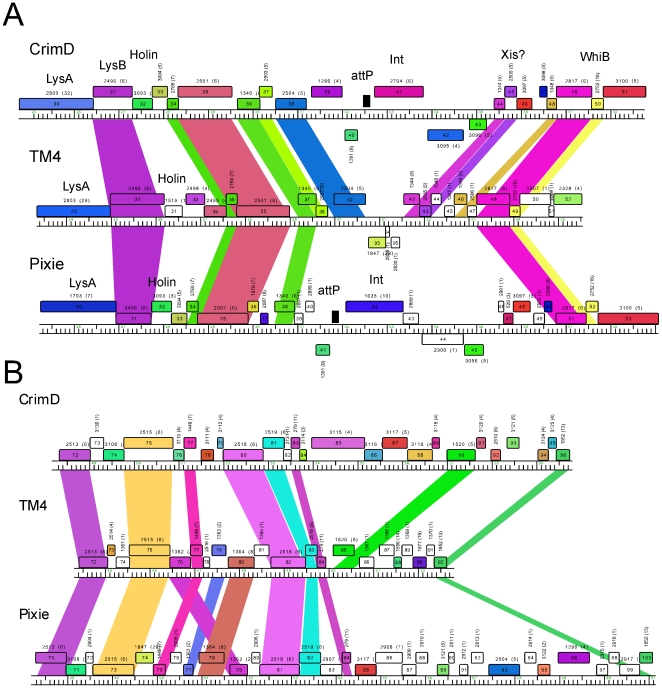
Putative deletions giving rise to phage TM4. Comparison of the TM4 genome to the other Cluster K genomes reveals two segments that appear to have been lost from TM4, and which may contribute to its non-temperate phenotype. **A**. The central parts of the CrimD, TM4, and Pixie genomes are aligned, with the colored shading reflecting the presence of genes of shared phamilies (i.e. homologues; note this shading does not reflect nucleotide sequence similarity as in Fig. 5). Although nucleotide sequence similarity is minimal, the alignment of shared genes suggests the loss of about 3.5 kbp form TM4 compared to its relatives. **B**. Alignment of the right ends of the CrimD, TM4 and Pixie genomes suggesting loss of ∼3.3 kbp from TM4 compared to its relatives; shading is as described for A.

Because none of the other phages are closely related to TM4 at the nucleotide sequence level (Fig. 5), the most informative comparisons emerge from comparing shared genes with amino acid sequence similarity (Fig. 11). We have focused on two regions of the genomes. The first is at the center of the genomes where the integration cassettes are found in the Subcluster K1 and K3 phages (Fig. 11A). TM4 genes *40* and *42* correspond to CrimD genes *38* and *44* such that the three leftwards transcribed TM4 genes, *93*, *94*, and *95*, occupy the location corresponding to CrimD genes *39* and *44* (Fig. 11A). Thus a simple explanation is that TM4 has lost a DNA segment approximately 3.5 kbp in length from a temperate parent that included the integrase gene and *attP* site. Interestingly, TM4 retains the predicted Xis function encoded by gene *43*, consistent with this interpretation (Fig. 11A).

The second region of interest is at the right end. The comparison between CrimD and TM4 is perhaps the most informative. CrimD contains homologues of TM4 gp84 and gp85 (CrimD gp83 and gp90), but they are separated by a 3.3 kbp DNA segment containing six predicted open reading frames (Fig. 11B). This suggests that TM4 has undergone a deletion of approximately 3.3 kbp between genes *84* and *85* from its putative temperate parent. It is plausible that one of the lost genes corresponds to a phage repressor, consistent with TM4's clear plaque phenotype. We note that the L5 repressor (gp71) is encoded near the right end of its genome, so this is a not an unusual genomic position for a repressor gene. Although none of the genes in these regions of the Cluster K1 or K3 genomes have sequence similarity to known repressors, all the K1 and K3 phages are homoimmune and are thus expected to share similar repressors. Pixie is quite different from the K1 genomes in this region, and there is only a single gene that they share in this interval, corresponding to Pixie gp85, Anaya gp90, Adephagia gp86, CrimD gp87, and Angelica gp84. However, preliminary analysis suggests that expression of CrimD gp87 from a plasmid in the host cell does not confer immunity to any of Cluster K phages and it is therefore an unlikely repressor candidate.

**Figure 12:**
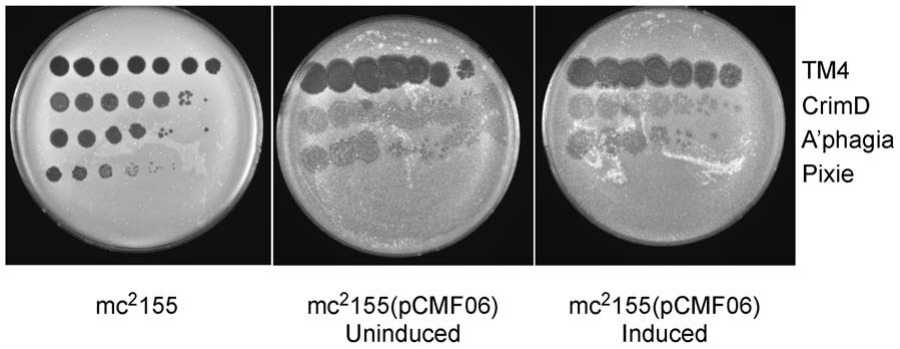
Expression of Pixie gp81 interferes with infection by Cluster K phages. *M. smegmatis* strains containing plasmid pCMF06 in which Pixie gene *81* is under control of the acetamidase-inducible promoter were tested for infection by Cluster K phages by spotting serial dilutions onto lawns of induced or uninduced strains. Expression of Pixie gp81 strongly inhibits Pixie infection and confers greater turbidity of other Cluster K phages.

All of the Cluster K genomes contain a member of Pham2518 with putative DNA binding motifs. We therefore tested whether a member of this phamily, Pixie *81* (Fig. 9) confers immunity to superinfection. Expression of Pixie gp81 strongly interferes with Pixie infection (Fig. 12), but has only modest effects on infection with TM4, CrimD, and Adephagia, supporting infection but yielding plaques with increased turbidity. If Pixie gp81 and its relatives encode phage repressors, we would expect to observe immunity to other Subcluster K1 and K3 phages; thus we propose that these proteins are involved in gene regulation but not as phage repressors. Unsuccessful attempts to delete Pixie *81* suggest it is likely to be an essential gene, consistent with this interpretation. TM4 gene *72* has a small internal deletion compared to its relatives but its functionality is unknown.

### Characterization of integration functions

**Figure 13:**
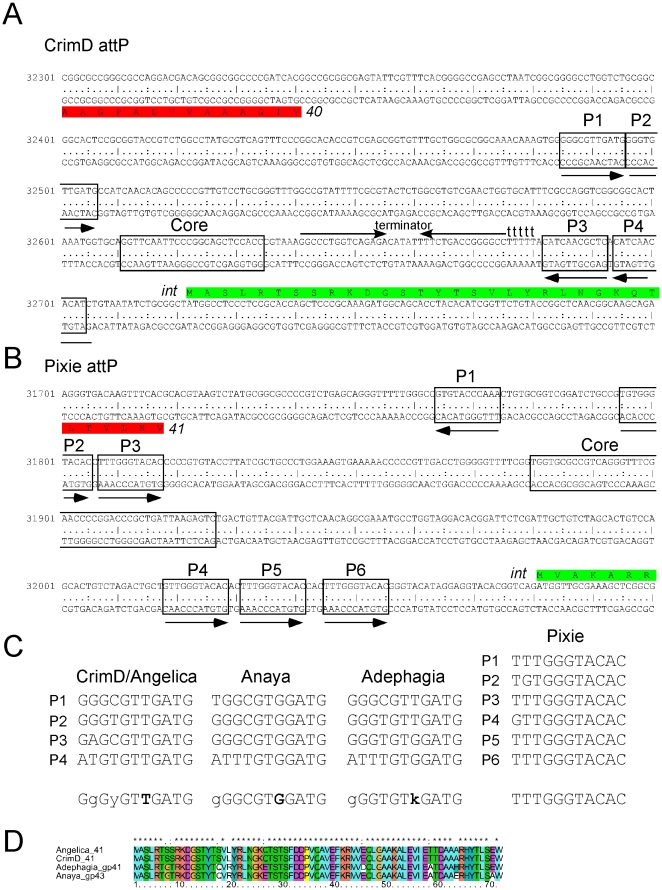
Organization of the *attP* sites of Cluster K genomes. **A**. Organization of the *attP* site of phage CrimD. The *attP* core region with sequence similarity to the chromosomal *attB* site is indicated, with predicted integrase-binding arm-type sites (P1, P2, P3, and P4) shown flanking it. The predicted orfs for integrase (*41*) and gene *40* are shown (see Fig. S2 for genomic context). A putative rightwards stem-loop terminator is located between the core and P3 sites; the *attP* sites for the other K1 phages are organized similarly. **B**. Organization of the *attP* site of phage Pixie, annotated as above. The K3 phage Pixie uses an integrase more distantly-related to those encoded by K1 phages, has a different *attP* core, and integrates into a different *attB* site. **C**. Alignment of the putative arm-type sites of Cluster K phages and the consensus sequences derived from them. Consensus positions shown in bold indicate differences between the K1 phages (CrimD, Angelica, Anaya, and Adephagia). **D**. Alignment of the N-terminal regions of the Subcluster K1 integrases that are predicted to recognize the arm-type sites.

The Cluster K1 mycobacteriophages are unusual in that they are predicted to integrate into a chromosomal *attB* site that overlaps the host tmRNA gene. Each contains a 24 bp common core segment corresponding to the extreme 3′ end of the tmRNA, suggesting that strand exchange occurs within or close to the segment corresponding to the tmRNA TψC stem [53]. The *M. tuberculosis* tmRNA gene differs from both *M. smegmatis* and the phages by a single base within the TψC loop, although this does not appear to interfere with integration since these phages form stable lysogens in *M. tuberculosis*. These *attP* common cores are located to the 5′ sides of the integrase genes in each of the K1 genomes (Fig. 13). A search for potential integrase arm-type DNA binding sites in CrimD reveals two pairs of 11 bp repeats, each flanking the common core (Fig. 13A–C), which we have labeled P1, P2, P3 and P4. Sites P3 and P4 are inverted in orientation relative to P1 and P2 (Fig. 13A); Anaya, Adephagia, and Angelica have similar organizations. We note that both mycobacteriophages Giles and L5 also contain pairs of putative arm-types [54], [55] although in these examples they are in direct orientation and L5 has several additional arm-type sites [54].

The CrimD four arm-type sites are not identical and vary in two positions (Fig. 13C). Angelica contains identical sites to CrimD, but interestingly both Anaya and Adephagia have potential arm-type binding sites with different consensus sequences (Fig. 13C). For example, whereas the consensus position 7 in CrimD is a T residue, in Anaya it is a G, and in neither case is there any departure from the consensus (Fig. 13C); in contrast, two of the Adephagia sites have T residues, and two have G residues. Because the arm-type sites are recognized and bound by the N-terminal domains of the tyrosine integrases we have compared these regions of the Subcluster K1 integrases (Fig. 13D). They are very closely related but contain amino acid substitutions at positions 17 and 19, which are thus candidates for involvement in recognition of the site features that differ between these genomes This is consistent with the model for arm-type site recognition by the lambda integrase [56]. An intriguing possibility is that Adephagia represents a transitional state between the evolution of a CrimD-type specificity and an Anaya-type specificity (Fig. 13). It is also interesting to note that these Cluster K1 phage integrases are close relatives of some of the Cluster F1 integrases, including Fruitloop gp40 (44% amino acid identity), although these integrate into a different *attB* site that overlaps a tRNA^ala^ gene (Msmeg_2138).

Because the putative Subcluster K1 *attB* site is distinct from those reported for other mycobacteriophages, this presents an opportunity to construct integration-proficient vectors that are compatible with those derived from L5 [29], Tweety [57], Giles [55], Bxb1 [58], and Ms6 [59]. To construct a new integration-proficient vector we PCR amplified a segment of the Adephagia genome containing the *int* gene (*41*) and *attP* site and inserted it into a mycobacterial non-replicating plasmid to generate pWHP02. Introduction of pWHP02 into electrocompetent *M. smegmatis* yielded transformants at a frequency of 5×10^5^ transformants/µg DNA. PCR analysis of four independent transformants showed that plasmid integration had occurred at the predicted *attB* site within the *M. smegmatis* tmRNA gene (data not shown).

The Subcluster K3 phage Pixie codes for an integrase more distantly related to the K1 integrases, although it shares substantial similarity to other mycobacteriophage integrases including the Tweety (Subcluster F1) integrase (44% amino acid identity) that was characterized previously [57]. The Pixie 47 bp *attP* common core (Fig. 13B) is similar to that of Tweety and they are predicted to integrate into the same *attB* site overlapping a tRNA^lys^ gene (Msmeg_4746). The Pixie *attP* site has an unusual array of potential arm-type binding sites with a pair to the left of the core (P2 and P3), and a set of three to the right (P4, P5, P6), all in direct orientation (Fig. 13B). A sixth site (P1) is located to the left of the core but oriented in the opposite orientation. These correspond closely to a consensus sequence with few departures (Fig. 13C). Phages with related integrases (e.g. Tweety) that use the same *attP* site do not share these arm-type sequences and presumably have different recognition specificities, although this remains ill-defined.

### Identification of Start Associated Sequences (SASs)

BlastN comparison of each of the Cluster K genomes against a database of all sequenced mycobacteriophage genomes reveals the presence of short repeated sequences located throughout the Cluster K genomes. The arrangement of these repeats is complex, and although their function is not known, their locations and orientations suggest a possible role in translation initiation. There are fundamentally two types of repeats. The first is a 13 bp asymmetric sequence present in between 11 and 19 copies in each Cluster K genome. The second is a pair of imperfect 17 bp inverted repeats located just upstream of a subset (about 50%) of the 13 bp repeats.

**Figure 14:**
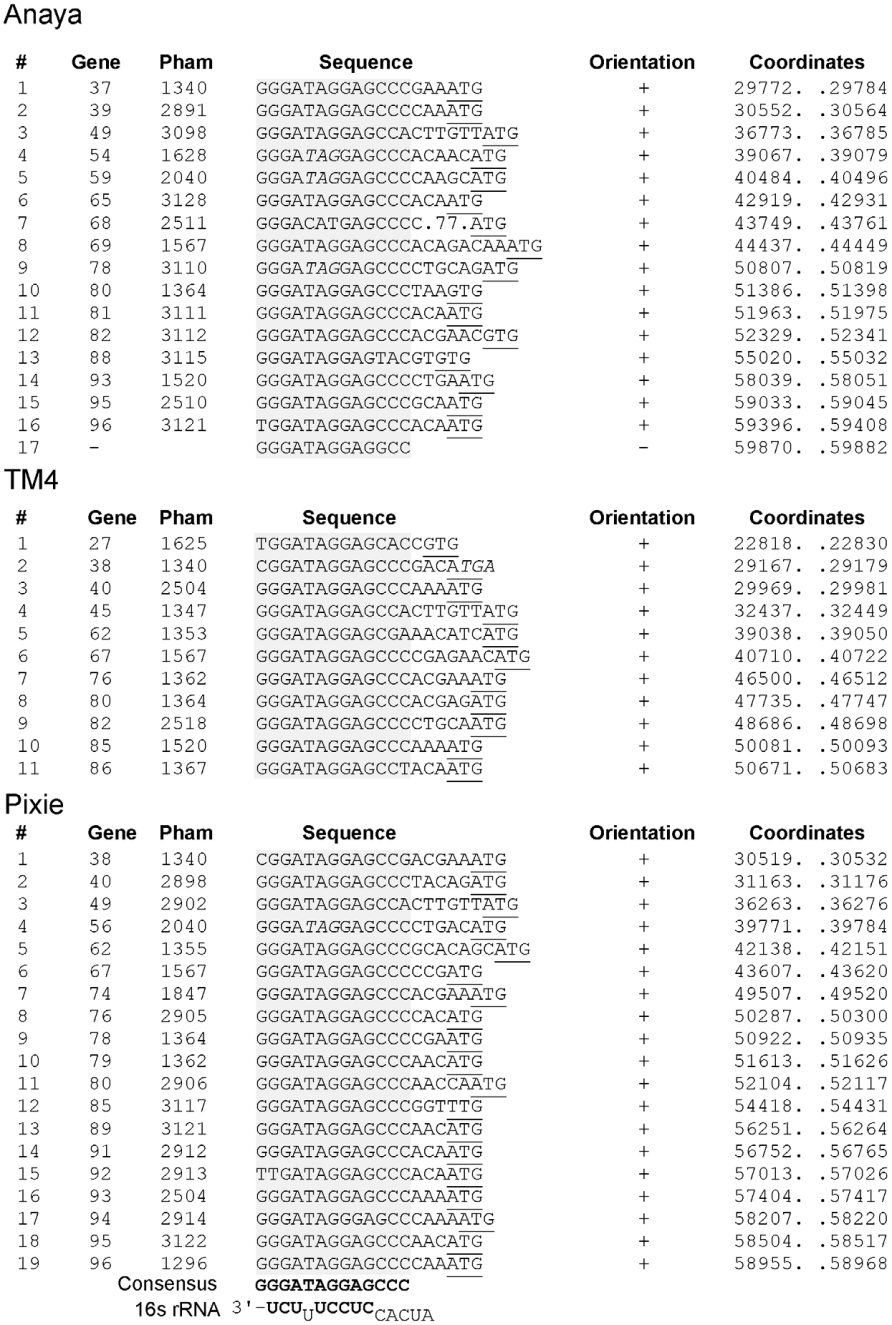
Location of Start Associated Sequences (SASs). Repeated sequences were identified in Cluster K phages through BLASTN comparisons with other mycobacteriophages, followed by scanning for the presence of the sequence 5′-GGGATAGGAGCCC, allowing for up to two deviations from the scanned sequence. (Pixie site #19 has three departures from the consensus but is included in the list because it is associated with an Extended SAS, see Fig. 15). Sites for Angelica, Adephagia, and CrimD are shown in Fig. S3. The sequence is asymmetric and most copies are orientated in one direction as indicated. With the rare exception of those sites in the opposite orientation (e.g. Anaya site #17), all are immediately upstream of gene start sites (Anaya site #7 is a notable exception.) The gene immediately downstream is listed along with its phamily (Pham) designation; the putative translation initiation codons are underlined; where the termination codon of the upstream gene lies within the conserved sequence it is italicized. The consensus sequence is shown in bold and the positions of the sites are shown by the colored highlighting. The extreme 3′ end of the 16S rRNA is shown with bases predicted to contribute to pairing with mRNA shown in bold. The genomic locations of these SASs are shown by numbered vertical arrows in Figs. 6, 7, 8, 9, S1 and S2).

The locations of the 13 bp sequence 5′-GGGATAGGAGCCC repeats are shown on the genome maps represented in Figures 6, 7, 8, 9, S1 and S2, and alignments of the sequences are shown in Figures 14 and S3. There are several striking features. First, it is apparent from the genome maps (Figs. 6, 7, 8, 9) that these sites are restricted to the right halves of the genomes containing non-structural protein genes. Second, virtually all of the repeats are located within a few nucleotides of the predicted translation start codons of downstream genes, typically 3–7 bp (Fig. 14, S3), and the start codon most commonly associated with SASs is ATG (80 of 93 sites identified) though ATG in general is only used by about 55% of mycobacteriophage genes. Third, the sequence is non-palindromic notwithstanding the symmetry of the outer parts of the sequence (i.e. 5′-GGGNNNNNNNCCC), and is typically present in one orientation only (Anaya, Adephagia, Angelica, and CrimD all have a single site in the opposite orientation that is not obviously associated with a gene start; Figs. 14 and S3). Fourth, these sequences are predominantly associated with genes that are separated from their upstream gene neighbors by more than 50 bp, relatively large intergenic regions within the context of typical phage genome organization (Tables S1, S2, S3, S4, S5, S6). Finally, this sequence is not common among mycobacteriophages, and outside of Cluster K genomes, only Corndog has a single copy with two deviations from the consensus. There is not a single copy of the consensus 13 bp sequence in *M. smegmatis* and only four when permitting a single deviation. Likewise there are no exact copies in *M. tuberculosis* H37Rv and only two with a single deviation.

This conserved sequence is in the position typically occupied by the Ribosome Binding Site (RBS). Indeed, the repeat contains the 5′-AGGAG sequence that is a core component of the Shine-Dalgarno sequence that pairs with the 3′ end of the 16S rRNA during translation initiation, and positions 2–4 of the conserved sequence have the capacity to extend the pairing with 16S rRNA (Fig. 14). However, it seems unlikely that this repeat simply corresponds to just a favorable translation initiation site. First, the starting base of the sequence is extremely well conserved (Fig. 14) but has no corresponding base to pair with in 16S rRNA. Second, positions 10–13 are also highly conserved, but do not have pairing potential with rRNA (Fig. 14). Nonetheless, the positioning of these repeats suggests a role in translation initiation – in contrast to the 13 bp stoperator sequences in L5 and other Cluster A phages that play a role in transcription regulation [60] – and we therefore propose that they be called Start Associated Sequences (SASs). Whether these act independently or represent binding sites for either a host- or phage-encoded gene product (either RNA or protein) remains to be determined. The conservation of these sites across the three subclusters – often associated with genes of different phamilies (Table 2) – strongly suggests that they play important roles for these phages.

**Table 2:** Conservation of mycobacteriophage gene phamilies containing SAS sequences in Cluster K phages.

PhagePham	1296	1340	1347	1353	1355	1362	1364	1367	1520	1567	1625	1628	1847	2040	2504	2510	2511	2518	2887	2891	2898	2902	2905	2906	2912	2913	2914	3098	3107	3110	3111	3112	3115	3117	3121	3123	3128
Anaya^1^	−	E	−	−	−	−	E	−	E	S	+	S	−	S	−	E	S	+	−	E	−	−	−	−	−	−	−	S	+	E	E	S	S	+	E	−	S
Angelica	E	E	−	−	−	−	−	−	E	S	+	S	−	S	E	E	S	+	−	−	−	−	−	−	−	−	−	S	S	E	E	S	S	+	E	S	−
Adephagia	−	E	−	−	−	−	E	−	E	S	+	S	−	S	E	E	S	+	E	−	−	−	−	−	−	−	−	S	S	E	E	S	S	+	E	−	−
CrimD	E	E	−	−	−	−	−	−	E	S	+	S	−	S	E	E	+	+	−	−	−	−	−	−	−	−	−	S	+	E	E	S	+	+	E	−	S
TM4	−	E	S	S	+	E	S		E	S	S	−	+	+	E	+	+	S	−	−	−	−	−	−	−	−	−	−	−	−	−	−	−	−	−	−	−
Pixie	E	E	−	−	S	E	E	−	−	S	+	+	S	S	E	−	+	+	−	−	S	S	S	S	E	S	E	+	+	−	−	−	−	S	E	−	−

1 Anaya, Angelica, Adephagia and CrimD all belong to Subcluster K1, TM4 to Subcluster K2, and Pixie to Subcluster K3.

S denotes that the genome contains a gene member of the designated phamily (pham) with an upstream SAS. E denotes ESAS's. + denotes that a phamily member is present, but there is no SAS or ESAS. − indicates that the genome does not contain a phamily member.

**Figure 15:**
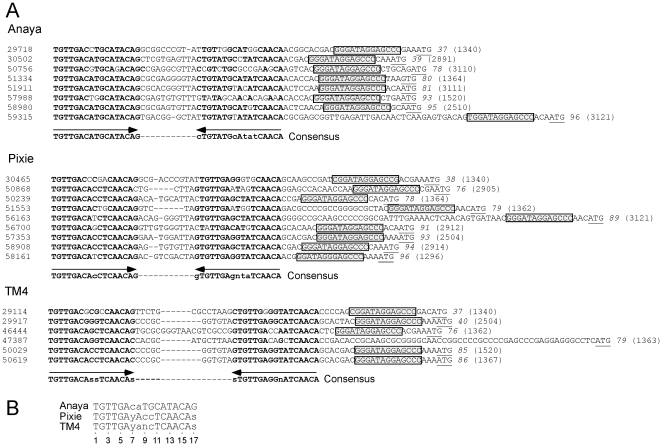
Extended SAS (ESAS) sequences. A subset of the SASs shown in Fig. 14 also contain a conserved sequence immediately upstream of the SAS (red arrows in Figs. 6, 7, 8, 9, S1, S2). These sequences contain a 17 bp imperfect inverted repeat separated by a variable spacer. **A**. Alignment of the extended SAS sequences for Anaya, Pixie, and TM4. The consensus sequence is shown with bases in upper case if there are two or fewer departures from the consensus, and in lower case if there are more than two departures but a greater than 50% agreement. **B**. Consensus sequences for each of the half sites within the extended SAS sequences. Upper case letters denote no more than four deviations from the consensus. Positions conserved 50% or more are shown in lower case letters. The SASs are indicated with the colored boxes and the putative start codon is underlined. The downstream gene is shown in italic type and its phamily designation is shown in parentheses. ESAS sequences for phages Adephagia, Angelica and CrimD are shown in Fig. S4; their 17 bp consensus sequences are very similar to their fellow Subcluster K1 page Anaya. A comparison of which phages genes are associated with SAS and ESAS sequences is shown in Table 2.

Approximately one half of the genes with an SAS also contain a second sequence feature composed of imperfect 17 bp inverted repeats (IRs) separated by a variable spacer (Figs. 15, S4). Because these are tightly associated with SASs, we refer to these as extended SASs (ESAS); in one notable exception the inverted repeat upstream of TM4 gene *79* does not appear to be associated with an SAS (Fig. 15A). For each genome a consensus sequence can be derived (Fig. 15B) from the left and right IRs, although the left IRs typically have a closer correspondence to the consensus than the right IRs (Figs. 15, S4); the spacer region between the IRs is variable, but is 4–13 bp in the vast majority of sites (Figs. 15A, S4). Interestingly, the consensus sequence of the IRs is different for phages of the three subclusters. The four Subcluster K1 phages have very similar IR consensus sequences (Figs. 15, S4), but differ from those of the Subcluster K2 (TM4) and K3 (Pixie) at positions 11, 12 and 13. For example, at position 11, there is predominantly a C in Anaya (in 15 of 16 IRs), but a T in both Pixie and TM4 (16 of 18 and 10 of 12 IRs respectively). At position 12, the C residue is strongly conserved in both Pixie and TM4, with no departures in any of the 30 constituent IRs, but this site is predominantly an A residue in Anaya (two of the 16 IRs have a C). At position 13 Pixie and TM4 have a consensus A residue, with no departures in any of the 30 IRs, whereas in Anaya this site is predominantly a T (two IRs have a G, and one has a A) (Fig. 15).

The ESAS sites are well conserved among the Cluster K genomes, in that if a gene of a particular phamily is associated with an ESAS in one genome, then other Cluster K genomes containing a gene member of that phamily also have an associated ESAS (Table 2). A notable exception is TM4 gene *80* (Pham 1364), which lacks an ESAS (it has an SAS), whereas all other phamily members have an ESAS (Table 2). Inspection of the TM4 sequence shows that the site is completely lacking, rather than having more highly diverged but related IRs. The conservation of these sites strongly suggests that they serve important functions for the phages, although it is not clear what they are. Because these are closely linked with the SASs that in turn are associated with translation initiation sites, it is tempting to assume that they also play a role in translation initiation. However, there is little support for the possibility that the two IRs form hairpin structures in mRNA, in that departures in the left and right IRs do not generally support RNA base-pairing. Therefore, it seems more likely that these represent binding sites for DNA-binding proteins and that the differences in consensus sequences represent different specificities in the three subclusters. One possible role might be in transcription initiation (i.e. promoters), but alternatively they could be operator sites for phage repressors. This latter explanation is attractive except that the K1 and K2 phages are homoimmune (Fig. 4), which is not consistent with the consensus differences. Furthermore, it is unclear why in virtually every occurrence the IRs are closely associated with translation initiation signals if they are operator sites. Finally, we note that in Pixie and TM4 each 17 bp IR itself has a symmetrical character, and can be considered as a 6 bp half site (5′-TGTTGA) separated by a 4 bp spacer from the inverse complement (Fig. 15B). However, this is not true for the Subcluster K1 phages because of the consensus differences at positions 11–13 (Fig. 15B, S4), as discussed above.

### Characterization of a conditionally-replicating mutant of TM4

Bardarov et al. (1997) described a conditionally replicating mutant of TM4 that fails to form plaques and fails to kill infected cells at temperatures of 37°C or above. This mutant – ph101 – is the basis for the construction of conditionally replicating shuttle phasmids used for delivery of reporter genes, transposons, and allelic exchange substrates to mycobacterial hosts [20], [23], [61]. The mutant was isolated using two rounds of hydroxylamine mutagenesis with the goal of isolating mutants that revert only at very low frequencies [20]. Because the functions of so few TM4 genes are known, we characterized the mutations in ph101.

Sequencing of the complete ph101 genome reveals a total of 23 differences (Table 3). One of these is a one base insertion in a non-coding region at the extreme right end of the genome; the others are all base substitution transitions, consistent with the mutagenic spectrum of hydroxylamine (Table 3). The large number of mutations reflects the heavy mutagenesis employed to recover the non-reverting mutants. Twelve of the base substitutions do not alter the predicted coding sequences, whereas the other ten do and are therefore candidates for contributing to the temperature-sensitive phenotype. Because the reversion frequency of ph101 is low (<10^−8^) it is likely that more than one mutation contributes to this phenotype. Three of the affected genes are predicted virion structure genes (*8*, *20*, *23*) and are unlikely to be involved in DNA replication (Fig. 6).

**Table 3:** Mutations in the ph101 genome relative to TM4.

Change #	Coordinates	Gene	Product (aa)	Codon change	aa change
1	2742	**5**	gp5 (501)	GCc-GCt	None
2	5519	**8**	gp8 (186)	aTC-gTC	**I46V**
3	8385	**13**	gp13 (139)	CAg-CAa	None
4	17199	**20**	gp20 (154)	cGC-tGC	**R116C**
5	20656	**23**	gp23 (784)	cCG-tCG	**P644S**
6	23751	**29**	gp29 (547)	AAc-AAt	None
7	27942	**36**	gp36 (394)	TAc-Tat	None
8	30039	**40**	gp40 (235)	gCC-aCC	**A19T**
9	31784	**42**	gp42 (118)	tCC-cCC	**S81P**
10	33509	**48**	gp48 (246)	GAa-Gag	None
11	33834	**48**	gp48 (246)	cCG-tCG	**P220S**
12	35899	**53**	gp53 (129)	GcT-GtT	**A126V**
13	39664	**63**	gp63 (101)	GCc-GCt	None
14	40515	**66**	gp66 (172)	gCC-aCC	**A131T**
15	40881	**67**	gp67 (100)	GcG-GtG	**A70A**
16	41761	**70**	gp70 (867)	GAa-GAg	None
17	45678	**75**	gp75 (303)	GCg-GCa	None
18	46889	**76**	gp76 (153)	CAc-CAt	None
19	47580	**79**	gp79 (103)	CAg-CAa	None
20	48991	**82**	gp82 (259)	TTc-TTt	None
21	49153	**82**	gp82 (259)	GAa-GAg	None
22	49824	**84**	gp84 (65)	gCG-aCG	**A30T**
23	52756			Ins 1 base	

**Figure 16:**
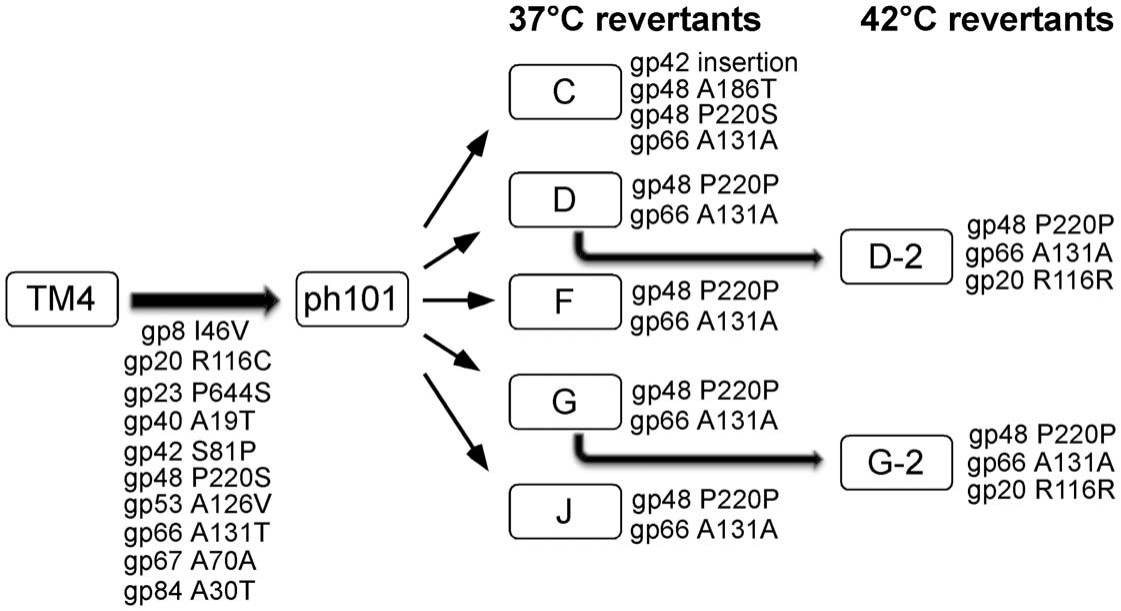
Mutations contributing to the conditionally replicating phenotype of TM4 mutant ph101. Phage ph101 is a temperate-sensitive conditionally replicating derivative of TM4 [20], and differs by more than 20 base substitutions, ten of which confer amino acid changes in predicted gene products as indicated. Five independent revertants capable of growth at 37°C were isolated, and the alleles at these ten positions were determined by sequencing of PCR amplicons (see Fig. 6). Four (D, F, G, and J) contain identical base changes that revert the mutations in genes *48* and *66* back to wild-type. One mutant (C) also contains the wild-type gene *66* sequence, but has a presumed intragenic suppressor mutation in gene *48*. Genes *48* and *66* thus contribute to the conditionally replicating phenotype. All of the revertants plate with reduced efficiency of plating at 42°C, and revertants growing normally at 42°C have an additional mutation reverting to the wild-type sequence in the putative tail gene, *20*.

To gain insight into which of the mutations contribute to the temperature sensitive phenotype we isolated five independent revertant mutants (C, D, F, G, and J) that are able to grow at 37°C, followed by PCR amplification and sequencing of the regions containing the ten non-synonymous mutations (Fig. 16). Revertants D, F, G, and J each contains nucleotide changes back to the wild-type sequence at mutations #10 and #14 (Table 3, Fig. 16), suggesting strongly that TM4 genes *48* and *66* contribute to the temperature-sensitive conditionally replicating phenotype. The involvement of gene *48* was somewhat surprising because the deletion in phasmid phAE159 removes the C-terminal 12 codons of gene *48* (see below). However, this region is poorly conserved among the TM4 gp48 relatives and is presumably not required for their function.

The fifth mutant (C) also has the reversion to wild-type sequence in gene *66*, but retains mutation #10 in gene *48*. However, it contains an additional mutation in codon 186 in gene *48* that presumably provides intragenic suppression of the first gene 48 mutation. This mutant also contains an apparent single base insertion at 31,784 within gene *42*, and although this is unlikely to contribute to the temperature sensitive phenotype, it suggests that *42* is not an essential gene. The specific roles of gp48 and gp66 are not known and neither has known non-mycobacteriophage homologues, but these data strongly suggest that both are required for normal replication of TM4.

All of the five mutants isolated at 37°C form plaques at 42°C with an efficiency of plating of approximately 10^−4^, suggesting that reversion of a third mutation is required to restore the full wild-type TM4 phenotype. Two independent mutants recovered at 42°C were analyzed as described above and both were found to contain a single additional base change that restores the wild type sequence at mutation #4 in gene 20 (Table 3, Fig. 16). Because gp20 is a putative virion structural protein (Fig. 6), the mutation in gene *20* is likely to contribute to the temperature-sensitive phenotype, but not to the conditional replicating property of ph101.

Sequencing of the cosmid-phage junctions in the shuttle phasmid phAE159 shows that a 5.8 kbp region between coordinates 33,877 and 39,722 is deleted and is therefore non-essential for TM4 growth (Fig. 6). This deletion removes all of genes *49* to *63* and the first sixteen codons of gene *64*, presumably rendering it non-functional. The deletion also removes the last 12 codons of gene *48*; but the extreme C-terminus of TM4 gp48 is not well conserved, and this modestly truncated product may retain its functionality. However, the proline residue altered in ph101 is absolutely conserved among all six related protein sequences.

### Concluding remarks

We have described here the genomes of the Cluster K group of mycobacteriophages, providing insights into the origins of the widely-used mycobacteriophage TM4, the genetic basis of a conditionally-replicating mutant of TM4, and a variety of enticing genomic features indicative of interesting but as yet not understood biological behavior. The presence of short repeated sequences suggests interesting regulatory features that have yet to be fully understood, but these also could be targets for homologous recombination and thus contribute to the mosaic nature of these genomes. The Cluster K phages clearly have a combination of features that make them particularly attractive for advances in tuberculosis genetics. First, all of the Cluster K phages infect both *M. tuberculosis* as well as *M. smegmatis* and appear to have relatively broad host ranges. Second, apart from TM4, all of them are temperate and form stable lysogens. Third, the genomes are relatively small – all are shorter than the average mycobacteriophage genome size of ∼70 kbp – and are amenable to manipulation using shuttle phasmid and recombineering strategies [62], [63].

## Materials and Methods

### Bacterial strains and Media

*M. smegmatis* mc^2^155, *Mycobacterium bovis* bacilli Calmette-Guérin (BCG) (Jacobs *et al.*, 1991; Snapper *et al.*, 1990) and *M. tuberculosis* mc^2^7000 have been described previously [64]. *M. marinum* strains M and 927 were kind gifts from Dr. Don G. Ennis, University of Louisiana, Lafayette, LA; *M. avium* 104 was a gift from Dr. William R. Bishai, Johns Hopkins School of Medicine, Baltimore, MD. Media were supplemented with carbenicillin (50 µg ml^−1^) and/or cycloheximide (10 µg ml^−1^) as required. *M. smegmatis* and *M. marinum* were grown in 7H9 liquid and on 7H10 plates; *M. tuberculosis*, *M. bovis*, and *M. avium* were grown in 7H9 liquid, and on 7H11 plates.

### Phage Isolation and purification

Phage Pixie was isolated from a dry soil sample obtained from a yard located in the Northwest Houston Metropolitan Area (TX, USA). Phage Anaya was isolated from a soil sample obtained from Grand Rapids, MI (MI, USA). Phage Adephagia was isolated from a soil sample obtained from Denton, TX (TX, USA). All phages were isolated by co-plating of soil extracts prepared with phage buffer (10 mM Tris/HCl pH 7.5, 10 mM MgSO_4_, 1 mM CaCl_2_, 68.5 mM NaCl), and *M. smegmatis*. The soil extract was filtered through a 0.22 µm filter. For Pixie, 50 µl of this sample was direct plated with 0.5 mL late-exponential-phase *M. smegmatis* mc^2^155 in 4.5 mL 0.35% mycobacterial top agar (MBTA) with 1 mM CaCl_2_. The MBTA/phage/bacterial mixture was distributed evenly on a plate of 7H10 agar (Difco) supplemented with carbenicillin, cycloheximide, 1 mM CaCl_2_ and 10% albumin dextrose complex (ADC). Phages Anaya and Adephagia were isolated by incubating 1 gram of soil with *M. smegmatis* mc^2^155 in 50 mL of LB plus 1 mM CaCl_2_ at 37°C with shaking for 24 hours. Remaining cells were then pelleted by centrifugation, and the supernatant was filtered through a 0.22 µm filter. Fifty microliters of the filtrate was co-plated on LB plates with 0.5 mL late-exponential phase *M. smegmatis* mc^2^155 in 4.5 mL LB top agar. All phages were incubated at 37°C, except for Anaya, which was incubated at 30°C. After several rounds of purification (Sarkis & Hatfull, 1998) high-titer stocks were prepared and used for subsequent studies.

### Genome Sequencing and Annotation

For phage Pixie, double-stranded DNA was phenol-extracted from dialyzed CsCl banded phage particles, then sequenced by 454 technology to 25-fold redundancy (∼4000 reads) at the University of Pittsburgh Genomics and Proteomics Core Laboratories as described previously [10]. Shotgun sequencing for phages Anaya and Adephagia was done by the Joint Genome Institute using both 454 (∼30-fold redundancy) and Illumina (∼100-fold redundancy) technologies. Reads were assembled with Newbler [65]; Consed [66] was used to assure quality control for the assemblies and identify the natures of the genome termini. To resolve remaining ambiguities, genome finishing was performed using targeted Sanger sequencing on phage genomic DNA templates. Finishing reads were incorporated into existing assemblies using PhredPhrap. The sequencing of TM4 [40], Angelica, and CrimD [8] was described previously. The Genbank accession numbers for Phages TM4, Pixie, Anaya, Adephagia, Angelica and CrimD are AF068845, JF937104, JF704106, JF704105, HM152764, and HM152767 respectively.

Finished sequences were analyzed and annotated in genome editors including DNAMaster (http://cobamide2.bio.pitt.edu), GBrowse [67], Apollo [68], and the University California Santa Cruz Genome Browser [69]; Glimmer [70], GeneMark [71], tRNA ScanSE [72], Aragorn [73], and Programmed Frameshift Finder [74] were used to identify genome features. Genes were assigned to phams, and genome maps and phamily circle diagrams were drawn using Phamerator, database Mycobacteriophage_83 (S.G.C., R.W.H., G.F.H., manuscript submitted). The threshold parameters of 32.5% identity with ClustalW and a BlastP E-value of 10^−50^, are different to those used previously, and were derived by optimizing pham assembly over a range of possible values (S.G.C., R.W.H., G.F.H., manuscript submitted). DotPlots were made using Gepard [75].

### Lysogen PCR Assays

Site-specific integration between the putative phage *attP* of Pixie, Angelica, Anaya, Adephagia, and CrimD with the corresponding *M. smegmatis attB* sites was confirmed in lysogens by PCR amplification of the *attL* and *attB* sites. Pelleted, potential lysogenic cells were suspended in 500 µl of 10 mM Tris (pH 8.0), 1 mM ethylenediaminetetraacetic acid (EDTA), heated for 20 minutes at 95°C and 10 µl was used in PCRs with *Pfu* polymerase (Stratagene), 5% Dimethyl sulfoxide (DMSO) and 10 nM dNTPs. Primers CMF1 and CMF2 were used to amplify bacterial *attB* of Pixie, primers CMF4 and CMF6 were used to amplify the bacterial *attB* of Angelica, CrimD, Adephagia and Anaya. Primers CMF3 and CMF2 were used to amplify *attL* of Pixie, primers CMF5 and CMF6 were used to amplify *attL* of Angelica, CrimD and Adephagia, and primers CMF4 and CMF13 were used to amplify *attL* of Anaya. Primer sequences are listed in Table S7.

### Immunity assays

Immunity to K cluster phages was tested by spotting serial dilutions of each phage onto lawns of *M. smegmatis* mc^2^155, mc^2^155(Pixie) lysogens, mc^2^155(Angelica) lysogens, mc^2^155(CrimD) lysogens, mc^2^155(Anaya) lysogens and mc^2^155(Adephagia) lysogens.

### Plasmid Constructions

Plasmid pWHP02 was constructed by amplifying the integrase gene (*41*) and *attP* site from Adephagia virions using the primers Ade-IntF and Ade-IntR. These primers added the restriction sites *SbfI* and *SacI* to flank the target region. The L5-derived integration vector pMH94 (KanR, *oriE*) was digested with *SbfI* and *SacI* to remove the L5 *attP* region, and the amplified, digested Adephagia *attP* region was ligated into the vector backbone. The ligation reaction was transformed into *E. coli* DH10B, and recovered colonies were picked into LB-Kan to isolate DNA. Correct plasmid sequences were confirmed through restriction digestion and sequencing. Plasmid pWHP02 was then transformed into electrocompetent *M. smegmatis* mc^2^155, and plated on 7H10 Kan. Recovered colonies were grown in 7H9 Kan, and used as a template in a PCR amplification to check for insertion into the tmRNA site using primers CMF4, CMF5 and CMF6.

Plasmid pCMF06 was constructed as follows. Two primers (CMF18 and CMF19) with *NdeI* and *HpaI* restriction sites were designed and used to amplify Pixie gene *81* from Pixie genomic DNA. This 899 bp fragment was inserted by sticky-end cloning into pLAM12. Plasmid pCMF06 contains Pixie gene *81* under the control of an acetamide inducible promoter, *oriE*, *oriM*, and a kanamycin-resistance gene. Primer sequences are listed in Table S7. The contributions of all authors are listed in Table S8.

## Supporting Information

Figure S1 **Genome map of Mycobacteriophage Angelica.** A map of the Angelica genome is shown with markers spaced at 100 bp intervals, with genes shown as colored boxes, either above (rightwards transcribed) or below (leftwards transcribed) the genome. Gene names are shown within the boxes, and the phamily number of that gene shown above with the number of phamily members in parentheses. Genes are colored according to their phamily, and white genes represent orphams (phams with only a single member). Vertical arrows with numbers show the positions of Start-Associated Sequences (SAS), either with (red arrows) or without (black arrows) extended SAS sequences (ESAS, see Figs. 14 and 15).
(PDF)

Figure S2 **Genome map of Mycobacteriophage CrimD.** A map of the CrimD genome is shown with markers spaced at 100 bp intervals, with genes shown as colored boxes, either above (rightwards transcribed) or below (leftwards transcribed) the genome. Gene names are shown within the boxes, and the phamily number of that gene shown above with the number of phamily members in parentheses. Genes are colored according to their phamily, and white genes represent orphams (phams with only a single member). Vertical arrows with numbers show the positions of Start-Associated Sequences (SAS), either with (red arrows) or without (black arrows) extended SAS sequences (ESAS, see Figs. 14 and 15).
(PDF)

Figure S3 **Location of Start Associated Sequences (SASs) in Adephagia, Angelica and CrimD.** Repeated sequences were identified in Adephagia, Angelica and CrimD genomes as described in Fig. 14. All three genomes contain a single site on the complementary strand.
(PDF)

Figure S4 **Extended SAS (ESAS) sequences in Adephagia, Angelica and CrimD.** **A**. ESAS sequences in the Angelica, Adephagia and CrimD genomes are shown as described in Figure 15. **B**. Consensus sequences for each of the half sites within the extended SAS sequences. Upper case letters denote no more than four deviations from the consensus. Positions conserved 50% or more are shown in lower case letters.
(PDF)

Table S1 **Revised gene coordinates for mycobacteriophage TM4.**
(PDF)

Table S2 **Gene coordinates for mycobacteriophage Anaya.**
(PDF)

Table S3 **Gene coordinates for mycobacteriophage Adephagia.**
(PDF)

Table S4 **Gene coordinates for mycobacteriophage Pixie.**
(PDF)

Table S5 **Gene coordinates for mycobacteriophage Angelica.**
(PDF)

Table S6 **Gene coordinates for mycobacteriophage CrimD.**
(PDF)

Table S7 **Primers used in this study.**
(PDF)

Table S8 **Author contributions.**
(XLSX)
