# Supplementary figures and images for: Cluster K Mycobacteriophages: Insights into the Evolutionary Origins of Mycobacteriophage TM4
Source: PLoS One. 2011 Oct 28;6(10):e26750. doi: 10.1371/journal.pone.0026750 (PMC3203893; doi:10.1371/journal.pone.0026750)

# Angelica

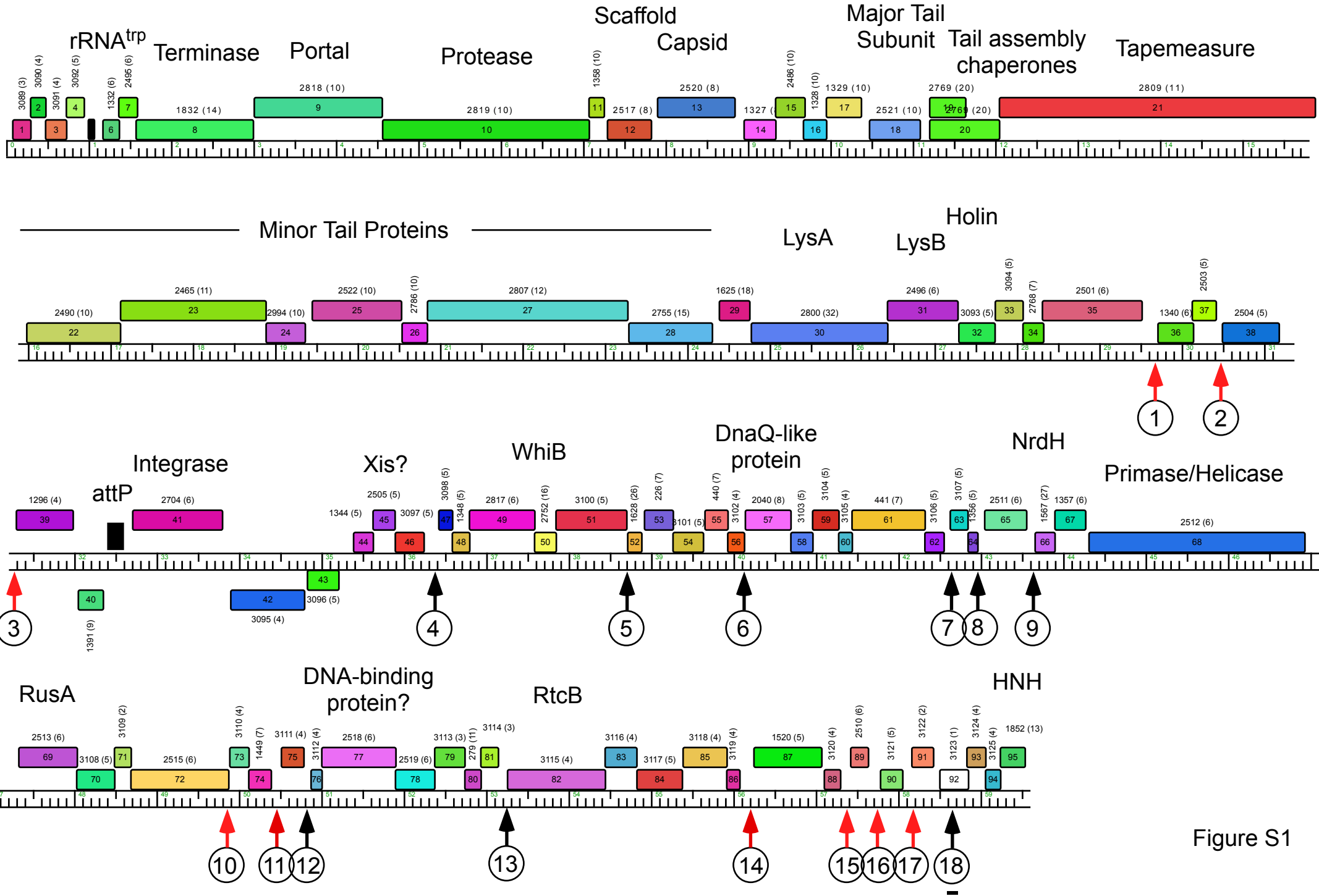

Figure S1

Supplement: Figure S1 — Genome map of Mycobacteriophage Angelica. A map of the Angelica genome is shown with markers spaced at 100 bp intervals, with genes shown as colored boxes, either above (rightwards transcribed) or below (leftwards transcribed) the genome. Gene names are shown within the boxes, and the phamily number of that gene shown above with the number of phamily members in parentheses. Genes are colored according to their phamily, and white genes represent orphams (phams with only a single member). Vertical arrows with numbers show the positions of Start-Associated Sequences (SAS), either with (red arrows) or without (black arrows) extended SAS sequences (ESAS, see Figs. 14 and 15). (PDF) [file pone.0026750.s001.pdf]

# CrimD

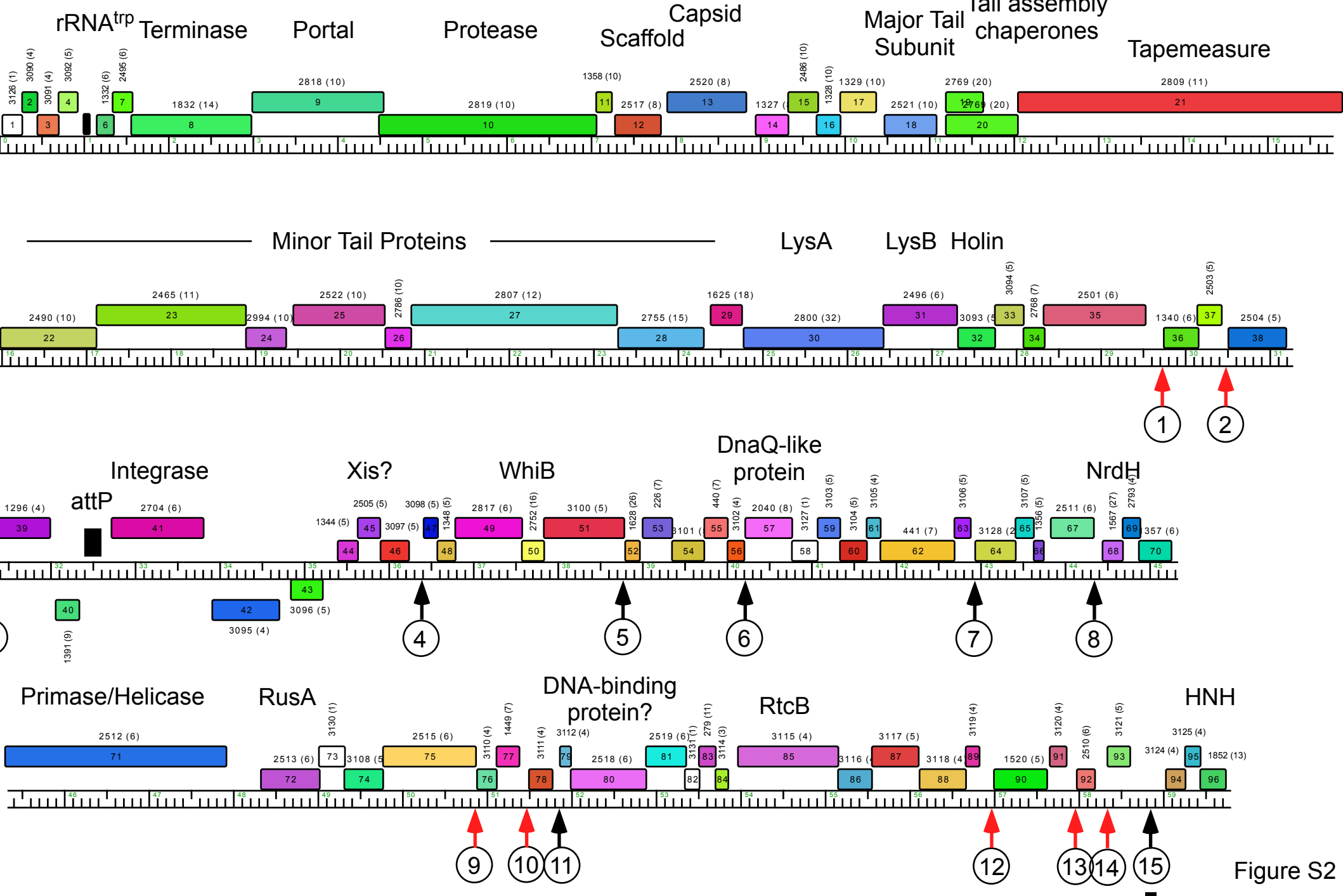

Figure S2

Supplement: Figure S2 — Genome map of Mycobacteriophage CrimD. A map of the CrimD genome is shown with markers spaced at 100 bp intervals, with genes shown as colored boxes, either above (rightwards transcribed) or below (leftwards transcribed) the genome. Gene names are shown within the boxes, and the phamily number of that gene shown above with the number of phamily members in parentheses. Genes are colored according to their phamily, and white genes represent orphams (phams with only a single member). Vertical arrows with numbers show the positions of Start-Associated Sequences (SAS), either with (red arrows) or without (black arrows) extended SAS sequences (ESAS, see Figs. 14 and 15). (PDF) [file pone.0026750.s002.pdf]
