# Supplementary material for: Cluster K Mycobacteriophages: Insights into the Evolutionary Origins of Mycobacteriophage TM4
Source: PLoS One. 2011 Oct 28;6(10):e26750. doi: 10.1371/journal.pone.0026750 (PMC3203893; doi:10.1371/journal.pone.0026750)
Supplement: Figure S3 — Location of Start Associated Sequences (SASs) in Adephagia, Angelica and CrimD. Repeated sequences were identified in Adephagia, Angelica and CrimD genomes as described in Fig. 14. All three genomes contain a single site on the complementary strand. (PDF) [file pone.0026750.s003.pdf]

Angelica

#	Gene	Pham	Sequence	Orientation	Coordinates
1	36	1340	GGGATAGGAGCCCGAAATG	+	29694. .29706
2	38	2504	GGGATAGGAGCCCACGATG	+	30476. .30488
3	39	1296	GGGATAGGAGCCCGAAATG	+	31280. .31292
4	47	3098	GGGATAGGAGCCACTTGTTATG	+	36403. .36415
5	52	1628	GGGATAGGAGCCCACAACATG	+	38697. .38709
6	57	2040	GGGATAGGAGCCCAAGCATG	+	40124. .40136
7	63	3107	GGGATAGGAGCCCACACCATGAGCAGCGAGGCAGGTCAGTG	+	42591. .42603
8	65	2511	GGGACATGAGCCCCC.70.TGAACAATG	+	42955. .42967
9	66	1567	GGGATAGGAGCCCACCGAAAAATG	+	43636. .43648
10	73	3110	GGGATAGGAGCCCTTGCAGATG	+	49869. .49881
11	75	3111	CCGATAGGAGCCCACAATG	+	50497. .50509
12	76	3112	GGGATAGGAGCCCACGAACGTG	+	50856. .50868
13	82	3115	GGGATAGGAGTACGTGTG	+	53243. .53255
14	87	1520	GGGATAGGAGCCCCAAATG	+	56233. .56245
15	89	2510	GGGATAGGAGCCCCAAAATG	+	57404. .57416
16	90	3121	TGGATAGGAGCCCACGATG	+	57769. .57781
17	91	3122	GGGATAGGAGCCCAAAATG	+	58147. .58159
18			GGGATAGGAGGCC	-	58634. .58646

Adephagia

#	Gene	Pham	Sequence	Orientation	Coordinates
1	36	1340	GGGATAGGAGCCCGAAATG	+	29744. .29756
2	38	2504	GGGATAGGAGCCCACGATG	+	30526. .30538
3	39	2887	GGGATAGGAGCCCACAATG	+	31328. .31340
4	47	3098	GGGATAGGAGCCACTTGTTATG	+	36173. .36185
5	52	1628	GGGATAGGAGCCCACAACATG	+	38548. .38560
6	57	2040	GGGATAGGAGCCCCAAGCATG	+	39968. .39980
7	63	3107	GGGATAGGAGCCCACACCATGAGCGGCGAGGCAGGTCAGTG	+	42439. .42451
8	66	2511	GGGATAGGAGCCCACCGAAAAATG	+	43483. .43495
9	73	3110	GGGATAGGAGCCCCTGCAGATG	+	49749. .49761
10	75	1364	GGGATAGGAGCCCACAGTG	+	50327. .50339
11	76	3111	CCGATAGGAGCCCACAATG	+	50913. .50925
12	77	3112	GGGATAGGAGCCCACGAACGTG	+	51272. .51284
13	84	3115	GGGATAGGAGAACGTGTG	+	53811. .53823
14	89	1520	GGGATAGGAGCCCCCTGAATG	+	56816. .56828
15	91	2510	GGGATAGGAGCCCACAATG	+	57798. .57810
16	92	3121	TGGATAGGAGCCCACGATG	+	58161. .58173
17			GGGATAGGAGGCC	-	58682. .58694

CrimD

#	Gene	Pham	Sequence	Orientation	Coordinates
1	36	1340	GGGATAGGAGCCCCGAAATG	+	29711. .29723
2	38	2504	GGGATAGGAGCCCACGATG	+	30495. .30507
3	39	1296	GGGATAGGAGCCCACAATG	+	31296. .31308
4	47	3098	GGGATAGGAGCCACTTGTTATG	+	36396. .36408
5	52	1628	GGGATAGGAGCCCACAACATG	+	38780. .38792
6	57	2040	GGGATAGGAGCCCAAGCATG	+	40202. .40214
7	64	3128	GGGATAGGAGCCCACCATG	+	42923. .42935
8	68	1567	GGGATAGGAGCCCACCGAAAAATG	+	44422. .44434
9	76	3110	GGGATAGGAGCCCTTGCAATG	+	50865. .50877
10	78	3111	GGGATAGGAGCCCACAATG	+	51482. .51494
11	79	3112	GGGATAGGAGCCCACGAACGTG	+	51845. .51857
12	90	1520	GGGATAGGAGCCCCTGAATG	+	56981. .56993
13	92	2510	GGGATAGGAGCCCACGATG	+	57961. .57973
14	93	3121	TGGATAGGAGCCCACAATG	+	58324. .58336
15			GGGATAGGAGGCC	-	58834. .58846

Figure S3
