# Supplementary material for: Cluster K Mycobacteriophages: Insights into the Evolutionary Origins of Mycobacteriophage TM4
Source: PLoS One. 2011 Oct 28;6(10):e26750. doi: 10.1371/journal.pone.0026750 (PMC3203893; doi:10.1371/journal.pone.0026750)
Supplement: Figure S4 — Extended SAS (ESAS) sequences in Adephagia, Angelica and CrimD. A. ESAS sequences in the Angelica, Adephagia and CrimD genomes are shown as described in Figure 15. B. Consensus sequences for each of the half sites within the extended SAS sequences. Upper case letters denote no more than four deviations from the consensus. Positions conserved 50% or more are shown in lower case letters. (PDF) [file pone.0026750.s004.pdf]

A

Adephagia

```
29690   TGTTGACCTGCATACAGGTGGCC--CGTATTGTTGGCATGGCAACAACGGCACAACGGGATAGGAGCCCGAAATG 36 (1340)
30473   TGTTGACATGCATACAGTTCGCG-GGTTACTGTATGCATACCAACAACGCACACGGGATAGGAGCCCACGATG 38 (2504)
31275   TGTTGACATACATACACTCGACG-GGTTACTGTATGTACATCAACAACGCACACGGGATAGGAGCCCACAATG 39 (2887
49677   TGTTGACGCGCATACAGAA---------TGTGTTGACGTGCAGACAGCCGAGGCGTTACCGTCTGCGCCGAAGCAAGTCACGGGATAGGAGCCCCTGCAGATG 73 (3110)
50276   TGTTGACATGCATACAGCGTGAG-GGGTACTGTATGCATGTCAACACACACCGGGATAGGAGCCCACAGTG 75 (1364)
50852   TGTTGACATGCATACAGCCCACG-GGTTACTGTATGCATACCAACAACGCACTGACCACCTACCGATAGGAGCCCACAATG 76 (3111)
56769   TGTTGACGCACATACAACC-----AATGTGTTTGCATATCAACACACACCACGGGATAGGAGCCCCCTGAATG 89 (1520)
57745   TGTTGACATGCATACAGCGCGGGT-GTTACTGTATGCATGTCAACAACTCAACAGGGATAGGAGCCCACAATG 91 (2510)
58080   TGTTGACATGCATACAGCCACGG--GCTATTGTATGTATATCAACAGCGCGAGCGGTTGAGATTGACAACTCAAGAGTGACAGTGGATAGGAGCCCACGATG 92 (3121)

        TGTTGACaTGCATACAG------------cTGTatGcATnnCAACA Consensus
```

Angelica

```
29640   TGTTGACCTGCATACAGGCGGCCC-GTATTGTTGGCATGGCAACAACGGCACAACGGGATAGGAGCCCGAAATG 36 (1340)
30423   TGTTGACATGCATACAGTTCGCGGGTTACTGTATGCATACCAACAACGCACACGGGATAGGAGCCCACGATG 38 (2504)
31228   TGTTGACATACATACAGCCGACGGGTTACTGTATGCATAGCAACAATGCGAGGGGATAGGAGCCCGAAATG 39 (1296)
49797   TGTTGACGCGCATACAGAA--------GGTGTTGACGTGCAGACAGTCGAGGCGTTACCGTCTGCGCCGAAGCAAGTCACGGGATAGGAGCCCTTGCAGATG 73 (3110)
50436   TGTTGACATGCATACAGCACGCGGGTTACTGTATGCATACCAACAACGCGCTGACCACCTACCGATAGGAGCCCACAATG 75 (3111)
56186   TGTTGACGCACATACAACCGA------TGTGTTTGCATATCAACACACACCACGGGATAGGAGCCCCAAATG 87 (1520)
57351   TGTTGACATGCATACAGCGCTGGTGTTACTGTATGCATGTCAACAACTCAACAGGGATAGGAGCCCCAAAATG 89 (2510)
57687   TGTTGACATGCATACAGTCACGAGGCTATTGTATGTACATCAACAGCGCGAGCGGTTGAGATTGACAACTCAAGAGTGACAGTGGATAGGAGCCCACGATG 90 (3121)
58097   TGTATGTACGCATACAGTCGTGC---TACTGTATGTATAGCAACAACGCGAACGGGATAGGAGCCCAAAATG 92 (3123)

        TGTTGACatGCATACAG-----------cTGTaTGCATanCAACA Consensus
```

CrimD

```
26957   TGTTGACCTGCATACAGGC-GGCCCGTATTGTTGGCATGGCAACAACGGCACGACGGGATAGGAGCCCCGAAATG 36 (1340)
30442   TGTTGACATGCATACAGTTCGCGGGTTACTGTATGCACATCAACAACGCACACGGGATAGGAGCCCACGATG 38 (2504)
31244   TGTTGACATGCATACAGCAGTGTGGTTATTGTATGTACATCAACAACGCCACGGGATAGGAGCCCACAATG 39 (1296)
50793   TGTTGACACGCATACAGAA--------GGTGTTGACGTGCAGACAGCCGGGGCGTTACCGTCTGCGCCGAAGCAAGTCACGGGATAGGAGCCCTTGCAATG 76 (3110)
51429   TGTTGACATGCATACAGCACACGGGTTACTGTATGCATGTCAACAACGCACACGGGATAGGAGCCCACAATG 78 (3111)
56934   TGTTGACGCACATACAACC--GATGTGTTTGCATATCAACACACACCACGGGATAGGAGCCCCTGAATG 90 (1520)
57908   TGTTGACATGCATACAGCGCCGGTGTTACTGTATGCATGTCAACAACTCAACAGGGATAGGAGCCCACGATG 92 (2510)
58243   TGTTGACATGCATACAGCG-ACAGGCTATTGTATGTATATCAACAGCGCGAGCGGTTGAGATTGACAACTCAAGAGTGACAGTGGATAGGAGCCCACAATG 93 (3121)

        TGTTGACATGCATACAG-----------nTGTATGcAtrtCAACA Consensus
```

B

```
Adephagia   TGTTGACATgCATACAg
Angelica    TGTTGayATGCATACAg
CrimD       TGTTGAyaTGCATACAg
            . . . . . . . . .
            1 3 5 7 9 11 13 15 17
```

Figure S4
