# Supplementary material for: Cluster K Mycobacteriophages: Insights into the Evolutionary Origins of Mycobacteriophage TM4
Source: PLoS One. 2011 Oct 28;6(10):e26750. doi: 10.1371/journal.pone.0026750 (PMC3203893; doi:10.1371/journal.pone.0026750)
Supplement: Table S1 — Revised gene coordinates for mycobacteriophage TM4. (PDF) [file pone.0026750.s005.pdf]

Table S1. Revised gene coordinates for mycobacteriophage TM4

Gene	Product	Strand	Start	Stop	Length	Type	Functions	Spacing[1]	(E)SAS [2]
1	gp1	F	100	234	135	ORF			
2	gp2	F	236	448	213	ORF		1	
3	gp3	F	438	689	252	ORF		-11	
4	gp4	F	664	2088	1425	ORF	Terminase	-26	
5	gp5	F	2101	3606	1506	ORF	Portal	12	
6	gp6	F	3587	5116	1530	ORF	Protease	-20	
7	gp7	F	5113	5298	186	ORF		-4	
8	gp8	F	5384	5944	561	ORF	Scaffold	85	
9	gp9	F	5999	6916	918	ORF	Capsid	54	
10	gp10	F	7028	7414	387	ORF		111	
11	gp11	F	7414	7764	351	ORF		-1	
12	gp12	F	7745	8017	273	ORF		-20	
13	gp13	F	8014	8433	420	ORF		-4	
14	gp14	F	8590	9204	615	ORF	Major Tail subunit	156	
15	gp15	F	9314	9763	450	ORF	Tail Assembly Chaperone	109	
16	gp16	F	9314	10236	444	ORF	Tail Assembly Chaperone	-450	
17	gp17	F	10184	13873	3690	ORF	Tapemeasure	-53	
18	gp18	F	13970	15097	1128	ORF	Minor Tail Subunit	96	
19	gp19	F	15094	16851	1758	ORF	Minor Tail Subunit	-4	
20	gp20	F	16851	17315	465	ORF		-1	
21	gp21	F	17408	18484	1077	ORF	Minor Tail Subunit	92	
22	gp22	F	18489	18743	255	ORF		4	
23	gp23	F	18727	21081	2355	ORF	Minor Tail Subunit	-17	
24	gp24	F	21138	21422	285	ORF		56	
25	gp25	F	21422	22426	1005	ORF	Minor Tail Subunit	-1	
26	gp26	F	22441	22677	237	ORF		14	
27	gp27	F	22832	23212	381	ORF		154	SAS-1
28	gp28	F	23219	23344	126	ORF		6	
29	gp29	F	23341	24984	1644	ORF	Lys A	-4	
30	gp30	F	24984	26186	1203	ORF	Lys B	-1	
31	gp31	F	26207	26593	387	ORF	Holin	20	
33	gp33	F	26676	27095	420	ORF		82	
34	gp34	F	27085	27576	492	ORF		-11	
35	gp35	F	27573	27821	249	ORF		-4	
36	gp36	F	27808	28992	1185	ORF		-14	
38	gp38	F	29183	29587	405	ORF		190	ESAS-2
39	gp39	F	29584	29844	261	ORF		-4	
40	gp40	F	29985	30689	705	ORF		140	ESAS-3
93	gp93	R	30757	31140	384	ORF		67	
94	gp94	R	31137	31226	90	ORF		-4	
95	gp95	R	31264	31455	192	ORF		37	
42	gp42	F	31544	31900	357	ORF		88	
43	gp43	F	31897	32154	258	ORF	Putative Xis	-4	
44	gp44	F	32159	32380	222	ORF		4	
45	gp45	F	32456	32677	222	ORF		75	SAS-4
46	gp46	F	32674	32955	282	ORF		-4	
47	gp47	F	32969	33172	204	ORF		13	
48	gp48	F	33177	33917	741	ORF		4	
49	gp49	F	33917	34147	231	ORF	WhiB	-1	
50	gp50	F	34144	34833	690	ORF		-4	
51	gp51	F	34790	34903	114	ORF		-44	
52	gp52	F	34900	35526	627	ORF		-4	
53	gp53	F	35523	35912	390	ORF		-4	
54	gp54	F	35905	36183	279	ORF		-8	
55	gp55	F	36180	36278	99	ORF		-4	
56	gp56	F	36275	36364	90	ORF		-4	
57	gp57	F	36361	36894	534	ORF	DnaQ-like protein	-4	
58	gp58	F	36891	37715	825	ORF		-4	
59	gp59	F	37712	38569	858	ORF	Hyp	-4	
60	gp60	F	38566	38739	174	ORF		-4	
61	gp61	F	38736	38954	219	ORF		-4	
62	gp62	F	39057	39377	321	ORF		102	SAS-5
63	gp63	F	39374	39679	306	ORF		-4	

64	gp64	F	39676	39882	207	ORF		-4	
65	gp65	F	39937	40056	120	ORF		54	
66	gp66	F	40125	40643	519	ORF		68	
67	gp67	F	40730	40975	246	ORF	NrdH	86	SAS-6
68	gp68	F	40975	41142	168	ORF		-1	
69	gp69	F	41139	41513	375	ORF		-4	
70	gp70	F	41528	44131	2604	ORF	Primase/Helicase	14	
72	gp72	F	44414	45055	642	ORF	RusA	282	
73	gp73	F	45052	45219	168	ORF		-4	
74	gp74	F	45216	45545	330	ORF		-4	
75	gp75	F	45538	46449	912	ORF		-8	
76	gp76	F	46446	46907	462	ORF		-4	ESAS-7
77	gp77	F	46904	47173	270	ORF		-4	
78	gp78	F	47196	47336	141	ORF		22	
79	gp79	F	47488	47700	283	ORF		151	
80	gp80	F	47753	48331	579	ORF	SprT	52	SAS-8
81	gp81	F	48331	48663	333	ORF		-1	
82	gp82	F	48704	49483	780	ORF	Putative DNA-binding	40	SAS-9
83	gp83	F	49480	49743	264	ORF		-4	
84	gp84	F	49737	49934	198	ORF		-7	
85	gp85	F	50097	50567	471	ORF		162	ESAS-10
86	gp86	F	50687	51007	321	ORF		119	ESAS-11
87	gp87	F	51133	51462	330	ORF		125	
88	gp88	F	51459	51626	168	ORF		-4	
89	gp89	F	51636	51872	237	ORF		9	
90	gp90	F	51883	52179	297	ORF		10	
91	gp91	F	52192	52362	171	ORF		12	
92	gp92	F	52356	52637	282	ORF	HNH domain	-7	

[1]Spacing is the distance between the start codon and the end of the nearest upstream gene. Negative values indicate overlapping reading frames.

[2]SAS indicates whether the intergenic upstream regions contain a Start Associated Sequence (SAS) or both an SAS and as Extended Start Associated Sequence (ESAS). SAS's were identified by searching for the sequence 5'-GGGATAGGAGCCC allowing up to two mismatches. ESAS sites contain an additional inverted repeat upstream of SAS. Numbers correspond to sites shown in Figure 6.
