# Supplementary material for: Cluster K Mycobacteriophages: Insights into the Evolutionary Origins of Mycobacteriophage TM4
Source: PLoS One. 2011 Oct 28;6(10):e26750. doi: 10.1371/journal.pone.0026750 (PMC3203893; doi:10.1371/journal.pone.0026750)
Supplement: Table S2 — Gene coordinates for mycobacteriophage Anaya. (PDF) [file pone.0026750.s006.pdf]

Table S2. Gene coordinates for Mycobacteriophage Anaya

Gene	Product	Strand	Start	Stop	Length	Type	Function	Spacing	(E)SAS [2]
1	gp1	F	85	297	213	ORF			
2	gp2	F	297	479	183	ORF		-1	
3	gp3	F	476	730	255	ORF		-4	
4	gp4	F	727	945	219	ORF		-4	
5	gp5	F	1001	1123	123	ORF		55	
6	tRNA Trp	F	1034	1111	78	tRNA	tRNA Trp (cca)	-90	
7	gp7	F	1212	1412	201	ORF		100	
8	gp8	F	1396	1632	237	ORF	Terminase small subunit	-17	
9	gp9	F	1616	3037	1422	ORF	Terminase large subunit	-17	
10	gp10	F	3049	4602	1554	ORF	Portal	11	
11	gp11	F	4547	7099	2553	ORF	Protease	-56	
12	gp12	F	7096	7281	186	ORF		-4	
13	gp13	F	7316	7855	540	ORF	Scaffold	34	
14	gp14	F	7935	8870	936	ORF	Capsid	79	
15	gp15	F	8982	9368	387	ORF		111	
16	gp16	F	9368	9721	354	ORF		-1	
17	gp17	F	9702	9983	282	ORF		-20	
18	gp18	F	9980	10405	426	ORF		-4	
19	gp19	F	10513	11124	612	ORF	Major Tail Subunit	107	
20	gp20	F	11244	11687	444	ORF	Tail Assembly Chaperone	119	
21	gp21	F	11244	12097	854	ORF	Tail Assembly Chaperone	-444	
22	gp22	F	12098	15934	3837	ORF	Tapemeasure	0	
23	gp23	F	16036	17172	1137	ORF	Minor Tail Subunit	101	
24	gp24	F	17173	18939	1767	ORF	Minor Tail Subunit	0	
25	gp25	F	18939	19415	477	ORF		-1	
26	gp26	F	19499	20581	1083	ORF	Minor Tail Subunit	83	
27	gp27	F	20588	20896	309	ORF		6	
28	gp28	F	20897	23329	2433	ORF	Minor Tail Subunit	0	
29	gp29	F	23452	24354	903	ORF		122	
30	gp30	F	24438	24809	372	ORF		83	
31	gp31	F	24827	26482	1656	ORF	LysA	17	
32	gp32	F	26479	27351	873	ORF	LysB	-4	
33	gp33	F	27362	27811	450	ORF	Holin	10	
34	gp34	F	27826	28152	327	ORF		14	
35	gp35	F	28149	28400	252	ORF		-4	
36	gp36	F	28387	29595	1209	ORF		-14	
37	gp37	F	29788	30219	432	ORF		192	ESAS-1
38	gp38	F	30204	30494	291	ORF		-16	
39	gp39	F	30568	30846	279	ORF		73	ESAS-2
40	gp40	F	30935	31222	288	ORF		88	
41	gp41	F	32164	32361	198	ORF		941	
42	gp42	R	32429	32707	279	ORF		67	
43	gp43	F	33089	34177	1089	ORF	Y-Integrase	381	
44	gp44	R	34279	35172	894	ORF		101	
45	gp45	R	35209	35589	381	ORF		36	
46	gp46	F	35748	36008	261	ORF		158	
47	gp47	F	36005	36271	267	ORF	Putative Xis	-4	
48	gp48	F	36273	36620	348	ORF		1	
49	gp49	F	36792	36959	168	ORF		171	SAS-3
50	gp50	F	36956	37168	213	ORF		-4	
51	gp51	F	37165	37956	792	ORF		-4	
52	gp52	F	37953	38216	264	ORF	WhiB	-4	
53	gp53	F	38213	39073	861	ORF		-4	
54	gp54	F	39085	39258	174	ORF		11	SAS-4
55	gp55	F	39287	39637	351	ORF		28	
56	gp56	F	39634	40017	384	ORF		-4	
57	gp57	F	40018	40299	282	ORF		0	
58	gp58	F	40296	40490	195	ORF		-4	
59	gp59	F	40502	41056	555	ORF	DnaQ- like protein	11	SAS-5
60	gp60	F	41056	41322	267	ORF		-1	
61	gp61	F	41319	41651	333	ORF		-4	
62	gp62	F	41648	41815	168	ORF		-4	
63	gp63	F	41812	42690	879	ORF		-4	

64	gp64	F	42687	42881	195	ORF		-4	
65	gp65	F	42935	43423	489	ORF		53	SAS-6
66	gp66	F	43420	43638	219	ORF		-4	
67	gp67	F	43635	43757	123	ORF		-4	
68	gp68	F	43839	44357	519	ORF		81	SAS-7
69	gp69	F	44458	44700	243	ORF	NrdH	100	SAS-8
70	gp70	F	44697	44936	240	ORF		-4	
71	gp71	F	44933	45304	372	ORF		-4	
72	gp72	F	45346	47955	2610	ORF	Primase/Helicase	41	
73	gp73	R	48244	48369	126	ORF		288	
74	gp74	F	48358	49056	699	ORF	RusA	-12	
75	gp75	F	49049	49510	462	ORF		-8	
76	gp76	F	49507	49710	204	ORF		-4	
77	gp77	F	49707	50813	1107	ORF		-4	
78	gp78	F	50826	51059	234	ORF		12	ESAS-9
79	gp79	F	51056	51328	273	ORF		-4	
80	gp80	F	51402	51863	462	ORF	SprT	73	ESAS-10
81	gp81	F	51979	52251	273	ORF		115	ESAS11
82	gp82	F	52348	52476	129	ORF		96	SAS-12
83	gp83	F	52473	53363	891	ORF	Putative DNA-binding	-4	
84	gp84	F	53360	53836	477	ORF		-4	
85	gp85	F	53830	54108	279	ORF		-7	
86	gp86	F	54108	54473	366	ORF		-1	
87	gp87	F	54470	54922	453	ORF		-4	
88	gp88	F	55035	56222	1188	ORF	RtcB	112	SAS-13
89	gp89	F	56222	56620	399	ORF		-1	
90	gp90	F	56620	57162	543	ORF		-1	
91	gp91	F	57155	57724	570	ORF		-8	
92	gp92	F	57721	57882	162	ORF		-4	
93	gp93	F	58056	58694	639	ORF		173	ESAS-14
94	gp94	F	58708	58926	219	ORF		13	
95	gp95	F	59049	59264	216	ORF		122	ESAS-15
96	gp96	F	59412	59681	270	ORF		147	ESAS-16
97	gp97	F	60067	60297	231	ORF		385	SAS-17[3]
98	gp98	F	60294	60476	183	ORF		-4	
99	gp99	F	60473	60775	303	ORF	HNH	-4	

[1] Spacing is the distance between the start codon and the end of the nearest upstream gene. Negative values indicate overlapping reading frames.

[2] SAS indicates whether the intergenic upstream regions contain a Start Associated Sequence (SAS) or both an SAS and as Extended Start Associated Sequence (ESAS). SAS's were identified by searching for the sequence 5'-GGGATAGGAGCCC allowing up to two mismatches. ESAS sites contain an additional inverted repeat upstream of SAS. Numbers correspond to sites shown in Figure 7.

[3] This SAS is on the complementary strand and may be associated a small leftwards-transcribed unassigned open reading frame.
