# Supplementary material for: Cluster K Mycobacteriophages: Insights into the Evolutionary Origins of Mycobacteriophage TM4
Source: PLoS One. 2011 Oct 28;6(10):e26750. doi: 10.1371/journal.pone.0026750 (PMC3203893; doi:10.1371/journal.pone.0026750)
Supplement: Table S3 — Gene coordinates for mycobacteriophage Adephagia. (PDF) [file pone.0026750.s007.pdf]

Table S3. Gene coordinates of mycobacteriophage Adephagia

Gene	Product	Strand	Start	Stop	Length	Type	Function	Spacing[1]	(E)SAS[2]
1	gp1	F	79	297	219	ORF			
2	gp2	F	294	479	186	ORF		-4	
3	gp3	F	476	730	255	ORF		-4	
4	gp4	F	727	954	228	ORF		-4	
5	tRNA-Trp	F	1003	1075	73	tRNA	tRNA Trp(cca)	48	
6	gp6	F	1180	1383	204	ORF		104	
7	gp7	F	1376	1603	228	ORF		-8	
8	gp8	F	1587	3008	1422	ORF	Terminase	-17	
9	gp9	F	3020	4579	1560	ORF	Portal	11	
10	gp10	F	4584	7094	2511	ORF	Protease	4	
11	gp11	F	7091	7276	186	ORF		-4	
12	gp12	F	7313	7855	543	ORF	Scaffold	36	
13	gp13	F	7931	8869	939	ORF	Capsid	75	
14	gp14	F	8981	9367	387	ORF		111	
15	gp15	F	9364	9720	357	ORF		-4	
16	gp16	F	9701	9982	282	ORF		-20	
17	gp17	F	9979	10404	426	ORF		-4	
18	gp18	F	10504	11118	615	ORF	Major Tail Subunit	99	
19	gp19	F	11232	11669	438	ORF	Tail Assembly Chaperone	113	
20	gp20	F	11232	12079	354	ORF	Tail Assembly Chaperone	-438	
21	gp21	F	12079	15915	3837	ORF	Tapemeasure	-1	
22	gp22	F	16017	17153	1137	ORF		101	
23	gp23	F	17154	18920	1767	ORF	Minor Tail Subunit	0	
24	gp24	F	18920	19396	477	ORF		-1	
25	gp25	F	19480	20562	1083	ORF	Minor Tail Subunit	83	
26	gp26	F	20569	20877	309	ORF		6	
27	gp27	F	20878	23310	2433	ORF		0	
28	gp28	F	23322	24335	1014	ORF		11	
29	gp29	F	24419	24790	372	ORF		83	
30	gp30	F	24808	26463	1656	ORF	Lys A	17	
31	gp31	F	26460	27332	873	ORF	Lys B	-4	
32	gp32	F	27343	27783	441	ORF	Holin	10	
33	gp33	F	27786	28124	339	ORF		2	
34	gp34	F	28121	28372	252	ORF		-4	
35	gp35	F	28359	29567	1209	ORF		-14	
36	gp36	F	29760	30191	432	ORF		192	ESAS-1
37	gp37	F	30176	30466	291	ORF		-16	
38	gp38	F	30542	31228	687	ORF		75	ESAS-2
39	gp39	F	31344	31742	399	ORF		115	ESAS-3
40	gp40	R	31816	32118	303	ORF		73	
41	gp41	F	32476	33564	1089	ORF	Integrase	357	
42	gp42	R	33669	34550	882	ORF		104	
43	gp43	R	34598	34978	381	ORF		47	
44	gp44	F	35138	35398	261	ORF		159	
45	gp45	F	35395	35661	267	ORF	Putative Xis	-4	
46	gp46	F	35663	36013	351	ORF		1	
47	gp47	F	36192	36359	168	ORF		178	SAS-4
48	gp48	F	36356	36568	213	ORF		-4	
49	gp49	F	36565	37356	792	ORF		-4	
50	gp50	F	37353	37616	264	ORF	WhiB	-4	
51	gp51	F	37613	38554	942	ORF		-4	
52	gp52	F	38566	38736	171	ORF		11	SAS-5
53	gp53	F	38768	39118	351	ORF		31	
54	gp54	F	39115	39498	384	ORF		-4	
55	gp55	F	39499	39777	279	ORF		0	
56	gp56	F	39774	39974	201	ORF		-4	
57	gp57	F	39986	40540	555	ORF	DnaQ- like protein	11	SAS-6
58	gp58	F	40540	40806	267	ORF		-1	
59	gp59	F	40803	41135	333	ORF		-4	
60	gp60	F	41132	41296	165	ORF		-4	
61	gp61	F	41293	42171	879	ORF		-4	
62	gp62	F	42168	42401	234	ORF		-4	
63	gp63	F	42477	42692	216	ORF		75	SAS-7

64	gp64	F	42714	42896	183	ORF		21	
65	gp65	F	42893	43402	510	ORF		-4	
66	gp66	F	43504	43746	243	ORF	NrdH	101	SAS-8
67	gp67	F	43743	43982	240	ORF		-4	
68	gp68	F	43979	44350	372	ORF		-4	
69	gp69	F	44392	47010	2619	ORF	Primase/Helicase	41	
70	gp70	F	47419	48117	699	ORF	RusA	408	
71	gp71	F	48110	48571	462	ORF		-8	
72	gp72	F	48568	49755	1188	ORF		-4	
73	gp73	F	49768	50001	234	ORF		12	ESAS-9
74	gp74	F	49998	50270	273	ORF		-4	
75	gp75	F	50343	50804	462	ORF	SprT	72	ESAS-10
76	gp76	F	50929	51204	276	ORF		124	ESAS-11
77	gp77	F	51291	51419	129	ORF		86	SAS-12
78	gp78	F	51416	52318	903	ORF	Putative DNA-binding	-4	
79	gp79	F	52315	52791	477	ORF		-4	
80	gp80	F	52785	53066	282	ORF		-7	
81	gp81	F	53066	53368	303	ORF		-1	
82	gp82	F	53368	53565	198	ORF		-1	
83	gp83	F	53562	53714	153	ORF		-4	
84	gp84	F	53826	55019	1194	ORF	RtcB	111	SAS-13
85	gp85	F	55016	55402	387	ORF		-4	
86	gp86	F	55402	55941	540	ORF		-1	
87	gp87	F	55949	56503	555	ORF		7	
88	gp88	F	56500	56661	162	ORF		-4	
89	gp89	F	56834	57463	630	ORF		172	ESAS-14
90	gp90	F	57495	57692	198	ORF		31	
91	gp91	F	57814	58029	216	ORF		121	ESAS-15
92	gp92	F	58177	58446	270	ORF		147	ESAS-16
93	gp93	F	58879	59109	231	ORF		432	SAS-17[3]
94	gp94	F	59103	59288	186	ORF		-7	
95	gp95	F	59285	59587	303	ORF	HNH endonuclease domain	-4	

[1]Spacing is the distance between the start codon and the end of the nearest upstream gene. Negative values indicate overlapping reading frames.

[2]SAS indicates whether the intergenic upstream regions contain a Start Associated Sequence (SAS) or both an SAS and as Extended Start Associated Sequence (ESAS). SAS's were identified by searching for the sequence 5'-GGGATAGGAGCCC allowing up to two mismatches. ESAS sites contain an additional inverted repeat upstream of SAS. Numbers correspond to sites shown in Figure 8.

[3]This SAS is on the complementary strand and may be associated a small leftwards-transcribed unassigned open reading frame.
