# Supplementary material for: Cluster K Mycobacteriophages: Insights into the Evolutionary Origins of Mycobacteriophage TM4
Source: PLoS One. 2011 Oct 28;6(10):e26750. doi: 10.1371/journal.pone.0026750 (PMC3203893; doi:10.1371/journal.pone.0026750)
Supplement: Table S4 — Gene coordinates for mycobacteriophage Pixie. (PDF) [file pone.0026750.s008.pdf]

Table S4. Gene coordinates of Mycobacteriophage Pixie

Gene	Product	Strand	Start	Stop	Length	Type	Notes	Spacing[1]	(E)SAS[2]
1	gp1	F	51	194	144	ORF			
2	gp2	F	191	415	225	ORF		-4	
3	gp3	F	412	579	168	ORF		-4	
4	gp4	F	576	716	141	ORF		-4	
5	gp5	F	834	1028	195	ORF		117	
6	gp6	F	1028	1255	228	ORF	Terminase Small subunit	-1	
7	gp7	F	1239	2663	1425	ORF	Terminase Large subunit	-17	
8	gp8	F	2674	4239	1566	ORF	Portal	10	
9	gp9	F	4178	6769	2592	ORF	Protease, capsid assembly	-62	
10	gp10	F	6766	6966	201	ORF		-4	
11	gp11	F	7042	7626	585	ORF	Scaffold	75	
12	gp12	F	7697	8620	924	ORF	Major Capsid Subunit	70	
13	gp13	F	8739	9122	384	ORF		118	
14	gp14	F	9122	9475	354	ORF		-1	
15	gp15	F	9456	9740	285	ORF		-20	
16	gp16	F	9737	10171	435	ORF		-4	
17	gp17	F	10319	10930	612	ORF	Major Tail Subunit	147	
18	gp18	F	11101	11535	435	ORF	Tail Assembly Chaperone	170	
19	gp19	F	11101	11945	846	ORF	Tail Assembly Chaperone	-435	
20	gp20	F	11949	16127	4179	ORF	Tapemeasure	3	
21	gp21	F	16222	17358	1137	ORF	Structural	94	
22	gp22	F	17358	19121	1764	ORF	Minor Tail Subunit	-1	
23	gp23	F	19121	19633	513	ORF		-1	
24	gp24	F	19682	20770	1089	ORF	Minor Tail Subunit	48	
25	gp25	F	20785	21102	318	ORF		14	
26	gp26	F	21086	23437	2352	ORF	Minor Tail	-17	
27	gp27	F	23437	23724	288	ORF		-1	
28	gp28	F	23724	24788	1065	ORF	Structural	-1	
29	gp29	F	24881	25237	357	ORF		92	
30	gp30	F	25256	26941	1686	ORF	LysA	18	
31	gp31	F	26938	27726	789	ORF	LysB	-4	
32	gp32	F	27737	28183	447	ORF	Holin	10	
33	gp33	F	28180	28527	348	ORF		-4	
34	gp34	F	28524	28775	252	ORF		-4	
35	gp35	F	28762	29892	1131	ORF		-14	
36	gp36	F	29889	30128	240	ORF		-4	
37	gp37	F	30167	30343	177	ORF		38	
38	gp38	F	30492	30932	441	ORF		148	ESAS- 1
39	gp39	F	30929	31120	192	ORF		-4	
40	gp40	F	31181	31366	186	ORF		60	SAS -2
41	gp41	R	31415	31717	303	ORF		48	
42	gp42	F	32081	33361	1281	ORF	Integrase (Y-Int)	363	
43	gp43	F	33361	33705	345	ORF		-1	
44	gp44	R	33786	34706	921	ORF		80	
45	gp45	R	34735	35157	423	ORF		28	
46	gp46	F	35491	35610	120	ORF	HTH DNA binding	333	
47	gp47	F	35607	35819	213	ORF	Putative Xis	-4	
48	gp48	F	35823	36200	378	ORF		3	
49	gp49	F	36282	36521	240	ORF		81	SAS -3
50	gp50	F	36518	36685	168	ORF		-4	
51	gp51	F	36777	37454	678	ORF		91	
52	gp52	F	37451	37720	270	ORF	WhiB	-4	
53	gp53	F	37717	39072	1356	ORF		-4	
54	gp54	F	39065	39484	420	ORF		-8	
55	gp55	F	39484	39777	294	ORF		-1	
56	gp56	F	39789	40346	558	ORF	DnaQ- like protein	11	SAS -4
57	gp57	F	40343	40612	270	ORF		-4	
58	gp58	F	40609	40917	309	ORF		-4	
59	gp59	F	40914	41795	882	ORF		-4	
60	gp60	F	41792	41968	177	ORF		-4	
61	gp61	F	41940	42161	222	ORF		-29	
62	gp62	F	42158	42433	276	ORF		-4	SAS -5
63	gp63	F	42430	42642	213	ORF		-4	

64	gp64	F	42642	42812	171	ORF		-1	
65	gp65	F	42812	42928	117	ORF		-1	
66	gp66	F	43008	43526	519	ORF		79	
67	gp67	F	43623	43865	243	ORF	NrdH	96	SAS -6
68	gp68	F	43865	44236	372	ORF		-1	
69	gp69	F	44279	46888	2610	ORF	Primase/Helicase	42	
70	gp70	F	47286	47972	687	ORF	RusA	397	
71	gp71	F	47965	48411	447	ORF		-8	
72	gp72	F	48408	48572	165	ORF		-4	
73	gp73	F	48565	49482	918	ORF		-8	
74	gp74	F	49525	49920	396	ORF		42	SAS -7
75	gp75	F	49917	50186	270	ORF		-4	
76	gp76	F	50303	50539	237	ORF		116	ESAS -8
77	gp77	F	50595	50807	213	ORF		55	
78	gp78	F	50938	51501	564	ORF	SprT	130	ESAS -9
79	gp79	F	51629	52024	396	ORF		127	ESAS 10
80	gp80	F	52122	52301	180	ORF		97	SAS -11
81	gp81	F	52298	53182	885	ORF		-4	
82	gp82	F	53179	53658	480	ORF		-4	
83	gp83	F	53658	54134	477	ORF		-1	
84	gp84	F	54143	54331	189	ORF		8	
85	gp85	F	54434	54901	468	ORF		102	SAS -12
86	gp86	F	54891	55484	594	ORF		-11	
87	gp87	F	55481	55792	312	ORF		-4	
88	gp88	F	55789	56052	264	ORF		-4	
89	gp89	F	56267	56524	258	ORF		214	ESAS -13
90	gp90	F	56524	56646	123	ORF		-1	
91	gp91	F	56768	56950	183	ORF		121	ESAS 14
92	gp92	F	57029	57292	264	ORF		78	SAS -15
93	gp93	F	57420	58109	690	ORF		127	ESAS 16
94	gp94	F	58224	58472	249	ORF		114	ESAS -17
95	gp95	F	58520	58786	267	ORF		47	SAS -18
96	gp96	F	58971	59669	699	ORF		184	ESAS 19
97	gp97	F	59831	60079	249	ORF		161	
98	gp98	F	60107	60334	228	ORF		27	
99	gp99	F	60346	60792	447	ORF		11	
100	gp100	F	60794	61081	288	ORF	HNH	1	

[1]Spacing is the distance between the start codon and the end of the nearest upstream gene. Negative values indicate overlapping reading frames.

[2]SAS indicates whether the intergenic upstream regions contain a Start Associated Sequence (SAS) or both an SAS and as Extended Start Associated Sequence (ESAS). The numbers correspond to those identified on the Pixie Genome Map, Figure 9.
