# Supplementary material for: Cluster K Mycobacteriophages: Insights into the Evolutionary Origins of Mycobacteriophage TM4
Source: PLoS One. 2011 Oct 28;6(10):e26750. doi: 10.1371/journal.pone.0026750 (PMC3203893; doi:10.1371/journal.pone.0026750)
Supplement: Table S5 — Gene coordinates for mycobacteriophage Angelica. (PDF) [file pone.0026750.s009.pdf]

Table S5. Gene coordinates of Mycobacteriophage Angelica

Gene	Product	Strand	Start	Stop	Length	Type	Notes	Spacing[1]	(E)SAS[2]
1	gp1	F	79	297	219	ORF			
2	gp2	F	294	479	186	ORF		-4	
3	gp3	F	476	730	255	ORF		-4	
4	gp4	F	727	945	219	ORF		-4	
5	tRNA-Trp	F	993	1066	74	tRNA	tRNA-Trp(cca)	47	
6	gp6	F	1171	1371	201	ORF		104	
7	gp7	F	1364	1591	228	ORF		-8	
8	gp8	F	1575	2996	1422	ORF	Terminase	-17	
9	gp9	F	3008	4561	1554	ORF	Portal	11	
10	gp10	F	4566	7073	2508	ORF	Protease	4	
11	gp11	F	7070	7255	186	ORF		-4	
12	gp12	F	7293	7826	534	ORF	Scaffold	37	
13	gp13	F	7902	8840	939	ORF	Capsid	75	
14	gp14	F	8952	9338	387	ORF		111	
15	gp15	F	9335	9691	357	ORF		-4	
16	gp16	F	9672	9953	282	ORF		-20	
17	gp17	F	9950	10375	426	ORF		-4	
18	gp18	F	10475	11089	615	ORF	Major Tail Subunit	99	
20	gp20	F	11203	12050	849	ORF	Tail Assembly Chaperone	113	
19	gp19	F	11203	11640	438	ORF	Tail Assembly Chaperone	-848	
21	gp21	F	12050	15883	3834	ORF	Tapemeasure	409	
22	gp22	F	15985	17121	1137	ORF		101	
23	gp23	F	17122	18888	1767	ORF	Minor Tail Subunit	0	
24	gp24	F	18888	19364	477	ORF		-1	
25	gp25	F	19448	20530	1083	ORF	Minor Tail Subunit	83	
26	gp26	F	20537	20845	309	ORF		6	
27	gp27	F	20846	23278	2433	ORF		0	
28	gp28	F	23290	24303	1014	ORF		11	
29	gp29	F	24387	24758	372	ORF		83	
30	gp30	F	24776	26431	1656	ORF	LysA	17	
31	gp31	F	26428	27282	855	ORF	LysB	-4	
32	gp32	F	27293	27733	441	ORF	Holin	10	
33	gp33	F	27736	28074	339	ORF		2	
34	gp34	F	28071	28322	252	ORF		-4	
35	gp35	F	28309	29517	1209	ORF		-14	
36	gp36	F	29710	30141	432	ORF		192	ESAS-1
37	gp37	F	30126	30416	291	ORF		-16	
38	gp38	F	30492	31181	690	ORF		75	ESAS -2
39	gp39	F	31296	31985	690	ORF		114	ESAS -3
40	gp40	R	32048	32350	303	ORF		62	
41	gp41	F	32708	33796	1089	ORF	Y-integrase	357	
42	gp42	R	33899	34792	894	ORF		102	
43	gp43	R	34829	35209	381	ORF		36	
44	gp44	F	35389	35628	240	ORF		179	
45	gp45	F	35625	35891	267	ORF		-4	
46	gp46	F	35893	36243	351	ORF		1	
47	gp47	F	36422	36589	168	ORF		178	SAS -4
48	gp48	F	36586	36798	213	ORF		-4	
49	gp49	F	36795	37586	792	ORF		-4	
50	gp50	F	37583	37846	264	ORF	WhiB	-4	
51	gp51	F	37843	38703	861	ORF		-4	
52	gp52	F	38715	38885	171	ORF		11	SAS-5
53	gp53	F	38918	39268	351	ORF		32	
54	gp54	F	39265	39633	369	ORF		-4	
55	gp55	F	39652	39930	279	ORF		18	
56	gp56	F	39927	40130	204	ORF		-4	
57	gp57	F	40141	40695	555	ORF	DnaQ- like protein	10	SAS-6
58	gp58	F	40695	40961	267	ORF		-1	
59	gp59	F	40958	41278	321	ORF		-4	

60	gp60	F	41275	41439	165	ORF		-4	
61	gp61	F	41436	42323	888	ORF		-4	
62	gp62	F	42320	42553	234	ORF		-4	
63	gp63	F	42629	42844	216	ORF		75	SAS-7
64	gp64	F	42844	42963	120	ORF		-1	
65	gp65	F	43046	43555	510	ORF		82	SAS-8
66	gp66	F	43657	43899	243	ORF	NrdH	101	SAS-9
67	gp67	F	43899	44270	372	ORF		-1	
68	gp68	F	44312	46930	2619	ORF	Primase/Helicase	41	
69	gp69	F	47327	48037	711	ORF	RusA	396	
70	gp70	F	48030	48491	462	ORF		-8	
71	gp71	F	48488	48691	204	ORF		-4	
72	gp72	F	48688	49875	1188	ORF		-4	
73	gp73	F	49888	50121	234	ORF		12	ESAS -10
74	gp74	F	50118	50390	273	ORF		-4	
75	gp75	F	50513	50788	276	ORF		122	ESAS -11
76	gp76	F	50875	51006	132	ORF		86	SAS 12
77	gp77	F	51003	51902	900	ORF		-4	
78	gp78	F	51899	52375	477	ORF		-4	
79	gp79	F	52375	52740	366	ORF		-1	
80	gp80	F	52740	52937	198	ORF		-1	
81	gp81	F	52934	53146	213	ORF		-4	
82	gp82	F	53258	54445	1188	ORF	RtcB	111	SAS-13
83	gp83	F	54442	54828	387	ORF		-4	
84	gp84	F	54828	55379	552	ORF		-1	
85	gp85	F	55387	55920	534	ORF		7	
86	gp86	F	55917	56078	162	ORF		-4	
87	gp87	F	56249	57070	822	ORF		170	ESAS -14
88	gp88	F	57102	57299	198	ORF		31	
89	gp89	F	57421	57636	216	ORF		121	ESAS-15
90	gp90	F	57785	58054	270	ORF		148	ESAS -16
91	gp91	F	58163	58426	264	ORF		108	ESAS-17
92	gp92	F	58503	58850	348	ORF		76	SAS-18
93	gp93	F	58831	59061	231	ORF		-20	
94	gp94	F	59058	59240	183	ORF		-4	
95	gp95	F	59237	59539	303	ORF	HNH	-4	

[1]Spacing is the distance between the start codon and the end of the nearest upstream gene. Negative values indicate overlapping reading frames.

[2]SAS indicates whether the intergenic upstream regions contain a Start Associated Sequence (SAS) or both an SAS and as Extended Start Associated Sequence (ESAS). Numbers correspond to sites shown in Supplemental Figure S1.
