# Supplementary material for: Cluster K Mycobacteriophages: Insights into the Evolutionary Origins of Mycobacteriophage TM4
Source: PLoS One. 2011 Oct 28;6(10):e26750. doi: 10.1371/journal.pone.0026750 (PMC3203893; doi:10.1371/journal.pone.0026750)
Supplement: Table S6 — Gene coordinates for mycobacteriophage CrimD. (PDF) [file pone.0026750.s010.pdf]

Table S6. Gene Coordinates of Mycobacteriophage CrimD

Gene	Product	Strand	Start	Stop	Length	Type	Function	Spacing[1]	(E)SAS[2]
1	gp1	F	39	272	234	ORF			
2	gp2	F	272	454	183	ORF		-1	
3	gp3	F	451	705	255	ORF		-4	
4	gp4	F	702	929	228	ORF		-4	
5	tRNA-Trp	F	978	1051	74	tRNA	tRNA-Trp(cca)	48	
6	gp6	F	1154	1357	204	ORF		102	
7	gp7	F	1341	1577	237	ORF		-17	
8	gp8	F	1561	2982	1422	ORF	Terminase	-17	
9	gp9	F	2994	4547	1554	ORF	Portal	11	
10	gp10	F	4492	7065	2574	ORF	Protease	-56	
11	gp11	F	7062	7247	186	ORF		-4	
12	gp12	F	7284	7826	543	ORF	Scaffold	36	
13	gp13	F	7902	8837	936	ORF	Capsid	75	
14	gp14	F	8949	9335	387	ORF		111	
15	gp15	F	9332	9688	357	ORF		-4	
16	gp16	F	9669	9950	282	ORF		-20	
17	gp17	F	9947	10372	426	ORF		-4	
18	gp18	F	10472	11086	615	ORF	Major Tail Subunit	99	
20	gp20	F	11199	12049	852	ORF	Tail Assembly Chaperone	112	
19	gp19	F	11199	11639	441	ORF	Tail Assembly Chaperone	-851	
21	gp21	F	12050	15886	3837	ORF	Tapemeasure	410	
22	gp22	F	15988	17124	1137	ORF	Minor Tail Subunit	101	
23	gp23	F	17125	18891	1767	ORF	Minor Tail Subunit	0	
24	gp24	F	18891	19367	477	ORF		-1	
25	gp25	F	19451	20533	1083	ORF	Minor Tail Subunit	83	
26	gp26	F	20540	20848	309	ORF		6	
27	gp27	F	20849	23281	2433	ORF	Minor Tail Subunit	0	
28	gp28	F	23293	24306	1014	ORF		11	
29	gp29	F	24388	24759	372	ORF		81	
30	gp30	F	24778	26433	1656	ORF	LysA	18	
31	gp31	F	26430	27302	873	ORF	LysB	-4	
32	gp32	F	27313	27753	441	ORF	Holin	10	
33	gp33	F	27750	28091	342	ORF		-4	
34	gp34	F	28088	28339	252	ORF		-4	
35	gp35	F	28326	29534	1209	ORF		-14	
36	gp36	F	29728	30159	504	ORF		121	ESAS -1
37	gp37	F	30144	30434	291	ORF		-16	
38	gp38	F	30511	31197	687	ORF		76	ESAS -2
39	gp39	F	31312	31998	687	ORF		114	ESAS -3
40	gp40	R	32062	32340	279	ORF		63	
41	gp41	F	32722	33810	1089	ORF	Y-integrase	381	
42	gp42	R	33914	34708	795	ORF		103	
43	gp43	R	34843	35220	378	ORF		134	
44	gp44	F	35399	35638	240	ORF		178	
45	gp45	F	35635	35901	267	ORF		-4	
46	gp46	F	35903	36241	339	ORF		1	
47	gp47	F	36415	36582	168	ORF		173	SAS -4
48	gp48	F	36576	36791	216	ORF		-7	
49	gp49	F	36788	37579	792	ORF		-4	
50	gp50	F	37576	37839	264	ORF	WhiB	-4	
51	gp51	F	37836	38786	951	ORF		-4	
52	gp52	F	38798	38971	174	ORF		11	SAS -5
53	gp53	F	39005	39355	351	ORF		33	
54	gp54	F	39352	39732	381	ORF		-4	
55	gp55	F	39733	40011	279	ORF		0	
56	gp56	F	40008	40208	201	ORF		-4	
57	gp57	F	40219	40773	555	ORF	DnaQ- like protein	10	SAS -6
58	gp58	F	40770	41075	306	ORF		-4	
59	gp59	F	41075	41341	267	ORF		-1	
60	gp60	F	41338	41658	321	ORF		-4	
61	gp61	F	41655	41819	165	ORF		-4	
62	gp62	F	41816	42694	879	ORF		-4	
63	gp63	F	42691	42885	195	ORF		-4	

64	gp64	F	42939	43418	480	ORF		53	SAS -7
65	gp65	F	43415	43630	216	ORF		-4	
66	gp66	F	43630	43749	120	ORF		-1	
67	gp67	F	43832	44341	510	ORF		82	
68	gp68	F	44443	44685	243	ORF	NrdH	101	SAS -8
69	gp69	F	44682	44891	210	ORF		-4	
70	gp70	F	44873	45259	387	ORF		-19	
71	gp71	F	45301	47919	2619	ORF	Primase/Helicase	41	
72	gp72	F	48327	49022	696	ORF	RusA	407	
73	gp73	F	49015	49320	306	ORF		-8	
74	gp74	F	49313	49774	462	ORF		-8	
75	gp75	F	49771	50871	1101	ORF	peptidase	-4	
76	gp76	F	50883	51116	234	ORF		11	ESAS -9
77	gp77	F	51113	51385	273	ORF		-4	
78	gp78	F	51498	51776	279	ORF		112	ESAS -10
79	gp79	F	51864	51995	132	ORF		87	SAS -11
80	gp80	F	51992	52885	894	ORF		-4	
81	gp81	F	52882	53358	477	ORF		-4	
82	gp82	F	53342	53515	174	ORF		-17	
83	gp83	F	53509	53706	198	ORF		-7	
84	gp84	F	53703	53855	153	ORF		-4	
85	gp85	F	53970	55157	1188	ORF	RtcB	114	
86	gp86	F	55154	55555	402	ORF		-4	
87	gp87	F	55555	56106	552	ORF		-1	
88	gp88	F	56117	56668	552	ORF		10	
89	gp89	F	56665	56826	162	ORF		-4	
90	gp90	F	56998	57627	630	ORF		171	ESAS 12
91	gp91	F	57659	57856	198	ORF		31	
92	gp92	F	57977	58192	216	ORF		120	ESAS -13
93	gp93	F	58340	58609	270	ORF		147	ESAS -14
94	gp94	F	59032	59262	231	ORF		422	SAS - 15
95	gp95	F	59259	59441	183	ORF		-4	
96	gp96	F	59438	59740	303	ORF	HnH	-4	

[1]Spacing is the distance between the start codon and the end of the nearest upstream gene. Negative values indicate overlapping reading frames.

[2]SAS indicates whether the intergenic upstream regions contain a Start Associated Sequence (SAS) or both an SAS and as Extended Start Associated Sequence (ESAS). Numbers correspond to sites shown in Supplemental Figure S2.
