# Supplementary material for: Cluster K Mycobacteriophages: Insights into the Evolutionary Origins of Mycobacteriophage TM4
Source: PLoS One. 2011 Oct 28;6(10):e26750. doi: 10.1371/journal.pone.0026750 (PMC3203893; doi:10.1371/journal.pone.0026750)
Supplement: Table S7 — Primers used in this study. (PDF) [file pone.0026750.s011.pdf]

Table S7. Primers used in this study.

Primer Name	Amplification Target	Sequence (5'-3')
CMF1	Pixie attB	TACCGCAGGCCATGAGACCGAACAGCAATG
CMF2	Pixie attB/attL	GTCGGGAGGTCCTATGGCACATGTGAGCGA
CMF3	Pixie attL	TCAGCCTTGAGCTTGTCCAGGGCTCGATC
CMF4	K1 attB	CACCGTAAGCGTCGTTGCAACCAATTAAGC-
CMF5	K1 attL (except Anaya)	CGGGTCAATGTCCTTGGCGACAACTTTC
CMF6	K1 attB/attL	GCGTGTACCTCACCAACTCGCTGTACT
CMF13	Anaya attL	CGGGTCAATGTCCTTGGCGACAACTTTC
AdeIntF	Adephagia integrase and attP	GCACACCGGCGAGCTCCGTTTGCT
AdeIntR	Adephagia integrase and attP	GACCTCCTGCAGGGCCCCAGATAC
CMF18	Pixie 81	aaaaaaacatatgGTGACCGCGCCGAGCAGGCACGGCGA
CMF19	Pixie 81	tttttttgttaacCGCCTGCGCCTCGATCGCGGGCTGCTC
DJS25	Ph101 Mutation Change 2F	GGTGATGCTGACGAGCTA
DJS26	Ph101 Mutation Change 2R	CCGAGTAAGCCTCTTGGAT
DJS27	Ph101 Mutation Change 4F	GGATTTGTTCTGATGCAACT
DJS28	Ph101 Mutation Change 4R	CGTCGATCATTTCCATAGTC
DJS29	Ph101 Mutation Change 5F	CGTCCGGTACGTGTTTGT
DJS30	Ph101 Mutation Change 5R	ATCGCATCCTCCAATGAC
DJS31	Ph101 Mutation Change 8F	GCAATCTGCTACACGAGG
DJS32	Ph101 Mutation Change 8R	CGTTTCTCGTTGGCTCTC
DJS33	Ph101 Mutation Change 9F	TTTCTGGACTAAACAATAGGCT
DJS34	Ph101 Mutation Change 9R	TGAGTTCATTGCGTCAG
DJS35	Ph101 Mutation Change 11F	TACGCCATGCAGTTCAAA
DJS36	Ph101 Mutation Change 11R	GTGTCGACTGCCCTTTGT
DJS37	Ph101 Mutation Change 12F	AGATTGACGCCGAGACAG
DJS38	Ph101 Mutation Change 12R	TCCTCAATTTCCTTGAGCTT
DJS39	Ph101 Mutation Change 14F	TCACGAAGGAAACACAGG
DJS40	Ph101 Mutation Change 14R	GGAGTTGCCGTGCTTATT
DJS41	Ph101 Mutation Change 22F	TGCAATGTCCAACCACAC
DJS42	Ph101 Mutation Change 22R	CGTACATCGGCAGGAACT
